# AMPK Activation by ENERGI Ameliorates Behavioral and Synaptic Deficits in a Mouse Model of Autism

**DOI:** 10.1007/s12035-025-05533-w

**Published:** 2025-11-27

**Authors:** Ming-Chia Chu, Chi-Chun Wu, Yueh-Jung Chung, Chieh-Yu Chang, Han-Fang Wu, Sze-Chi Tsai, Tzu-Ning Peng, Tzu-Jung Yang, Hui-Ching Lin

**Affiliations:** 1 Department and Institute of Physiology, College of Medicine, National Yang Ming Chiao Tung University, Taipei, Taiwan; 2 Department of Optometry, MacKay Medical College, New Taipei City, Taiwan; 3 Ph.D. Program in Medical Neuroscience, College of Medical Science and Technology, Taipei Medical University, Taipei, Taiwan; 4 Brain Research Center and Membrane Protein Structural Biology Research Center, National Yang Ming Chiao Tung University, Taipei, Taiwan

**Keywords:** AMP-activated protein kinase (AMPK), Autism spectrum disorder, Synaptic plasticity, Dendritic spine morphology, Valproate-induced autism model

## Abstract

Autism spectrum disorder (ASD), a neurodevelopmental disorder, is characterized by synaptic dysregulation as its underlying pathophysiological mechanism. AMP-activated protein kinase (AMPK), an intracellular energy sensor, plays a pivotal role in regulating synaptic integrity and function. Current treatments for ASD exhibit limited benefits in alleviating the core symptoms of ASD. Consequently, we investigated the therapeutic potential of ENERGI, a novel AMPK-activating compound, in a valproate (VPA)-induced mouse model of ASD. ENERGI was administered via drinking water to VPA-induced ASD offspring. After 7 days of treatment, ENERGI gradually alleviated social defects, repetitive behaviors, and emotional comorbidities in VPA-induced ASD offspring. At the synaptic level, ENERGI treatment restored aberrant plasticity, spine structure, and dendritic arborization in the hippocampus of VPA-induced ASD offspring. Notably, the curative effects of ENERGI in VPA-induced ASD offspring were equivalent to those of D-cycloserine (DCS), a known therapeutic candidate for ASD. Moreover, ENERGI demonstrated superior efficacy in restoring spine abnormalities than DCS. Mechanistically, 7-day ENERGI treatment reversed the reduction in AMPK phosphorylation, and normalized the elevated PSD95 and synaptic GluA2 levels in VPA-induced ASD offspring, whereas DCS treatment only rescued the synaptic GluA2 levels. Overall, these findings suggest that AMPK activation by ENERGI effectively reverses behavioral and synaptic deficits in a preclinical ASD model, supporting AMPK as a promising target for developing novel ASD therapies.

**Supplementary Information:**

The online version contains supplementary material available at 10.1007/s12035-025-05533-w.

## Introduction

Autism spectrum disorder (ASD) is a pervasive neurodevelopmental disorder characterized by the core symptoms of social difficulties and repetitive behaviors [1, 2], accompanied by various comorbidities [3]. The prevalence of ASD has dramatically increased, affecting approximately 1% of the population in recent decades [4]. Despite the identification of over 1000 associated genes [5] and various environmental risk factors, the heterogeneity of ASD continues to hinder effective drug development. Currently, about half of individuals with ASD are prescribed psychotropic medications that mostly address comorbid symptoms, rather than core symptoms [6]. The only two FDA-approved drugs for ASD, risperidone and aripiprazole, are both indicated for treating irritability [7–9]. Other clinical medications, including tricyclic antidepressants, selective serotonin reuptake inhibitors, and N-methyl-D-aspartate (NMDA) receptor antagonists, show limited efficacy for core ASD symptoms and often cause side effects [10–12]. These limitations highlight the urgent need for novel therapeutics that directly target core ASD symptoms.

Synaptic dysregulation has emerged as a central pathological mechanism in ASD. Evidence from both human and animal studies suggests that abnormalities in synaptic structure and function disrupt neuronal connectivity and synaptic plasticity, ultimately impairing brain network integrity and contributing to autistic behaviors [13–15]. However, ASD remains exclusively diagnosed based on behavioral criteria [16], reflecting a lack of robust biomarkers. Among emerging molecular targets, AMP-activated protein kinase (AMPK), a cellular energy sensor, has gained attention for its role in several brain disorders [17–19]. In the brain, AMPK is activated in response to increased cellular AMP/ATP ratios, and acts to restore energy homeostasis by promoting catabolic pathways [20, 21]. Synapses represent the most energy-consuming components of the brain [22, 23]. Notably, studies have shown that AMPK plays a pivotal role in sustaining neuronal energy levels during synaptic plasticity and transmission [24, 25]. AMPK activation has been reported to suppress neuronal polarization and axonal outgrowth under metabolic stress during development, while modulating synaptic remodeling in aging neurons [26–28]. Additionally, dysregulated AMPK signaling has been found to impair dendritic spine morphology and dendritic branching under pathological conditions [29, 30]. These findings position AMPK as a central mediator between cellular energy metabolism and synaptic regulation, suggesting its potential implication in ASD-related synaptopathology.

ENERGI, a purine analogue identified from bamboo (*Phyllostachys edulis*) shoot extract, was developed through a drug discovery platform [31]. Previous studies have demonstrated that ENERGI activates AMPK by dose-dependently increasing Thr172 phosphorylation [31, 32]. Its anti-tumor and anti-inflammatory effects in various disease models were AMPK-dependent and reversed by AMPK inhibition [31–33]. Considering its AMPK-activating properties, this study aimed to investigate the therapeutic potential of ENERGI for behavioral and synaptic deficits associated with ASD using a valproate (VPA)-induced mouse model. We examined ASD-related behaviors, neuronal structure, and synaptic plasticity following ENERGI treatment in VPA-induced offspring. D-cycloserine (DCS), an established ASD treatment, was applied to further evaluate the efficacy of ENERGI for ASD; the underlying molecular mechanism was also investigated.

## Materials and Methods

### Animals and Experimental Design

Experiments in this study were conducted according to the National Institute of Health Guide for the Care and Use of Laboratory Animals (USA) and approved by the Institutional Animal Care and Use Committee at National Yang Ming Chiao Tung University (Taiwan) with a project number 1091206n. Wild-type C57BL6 mice were purchased from the National Laboratory Animal Center (Taiwan). All animals were maintained in a room with a constant temperature of 24 ± 1 °C and humidity of 50 ± 5% under a 12/12 h light/dark cycle. Female mice were mated with male mice, and pregnancy were determined by the presence of the vaginal plug on embryonic day 0 (E0). Pregnant mice were received single intraperitoneal injection of sodium valproate (NaVPA, 500 mg/kg, Sigma-Aldrich, St. Louis, MO) on E12.5 [34–36]. The control group were injected with equal volume of sterile saline.

Dams were housed individually raising their own litters. All pups were weaned and housed in same-sex (3–4 pups/cage) on a postnatal day 21 (PND21). One to three male offspring were randomly selected from each litter to avoid litter effects. Male offspring were randomly assigned to vehicle, ENERGI (a proprietary compound generously provided by Energenesis Biomedical CO., LTD., Taipei, Taiwan), or DCS (Abcam, ab120121) treatment starting on PND28. The vehicle and ENERGI groups were further divided into subgroups receiving 3-, 5-, or 7-day treatments, with behavioral tests performed on PND31, PND33, PND35, respectively. Behavioral tests were conducted in the following order: three-chamber sociability test, marble burying test, open-field test, and elevated plus maze test, with a 4-h interval between each test. Assessments for synaptic plasticity, spine morphology, and molecular mechanisms were performed immediately after behavioral tests.

### Drug Preparation and Administration

ENERGI is a water-soluble compound with a water solubility of 1000 mg/L at room temperature. ENERGI was dissolved in boiled water to a final concentration of 60 mg/L, and administered to mice by drinking water at a target dose of 15 mg/kg/day. Mice were treated with water for control conditions. Pharmacokinetic data showed rapid absorption (peak plasma concentrations within 0.05–0.25 h in rats, IV/IP) and dose-dependent increases in systemic exposure. Toxicology data showed that 50 mg/kg/day caused renal changes in rats, which were absent or fully reversible at 15 mg/kg/day.

To minimize the inter-animal variations in ENERGI intake, two mice were housed per cage with a single bottle of ENERGI solution. Daily water intake per cage was recorded (Fig. S1d–f), and divided equally between two mice. Individual ENERGI intake was calculated based on each mouse’s body weight (Fig. S1a–c). Mice with estimated intake below 80% of the target dose (15 mg/kg/day) during the treatment period were excluded from following experiments.

For DCS treatment, animals were intraperitoneally injected with DCS (20 mg/kg/day), or with vehicle (saline) for control conditions once per day for 7 days from PND28 [37, 38].

### Three-chamber Sociability Test

Social behavior was assessed using the three-chamber sociability test [35]. The three-chamber apparatus was made of Plexiglas (30 × 60 × 30 cm) with two identical plastic cylinders respectively positioned on both side of the apparatus. The cylinders (8 cm in diameter, 10.5 cm in height) contain holes on their walls allowing mice sniffing with each other. Mice with similar age, sex, and weight to the test mouse, were served as stranger mice. The stranger mice had no previous physical contact with the test mouse, and were habituated to the plastic cylinders in the three-chamber apparatus for 30 min 24 h before the test. Before the sociability test, each mouse was placed into the center of the apparatus and freely explored for 5 min habituation. In the sociability session, one stranger mouse (stranger 1, S1) was enclosed in one of the plastic cylinders, and another cylinder was remained empty in the opposite side of the apparatus. The position of S1 was randomly assigned. If the test mouse showed side preference (> 180 s on one side) during habituation, stranger mouse was placed on the opposite side to counterbalance the bias. The side chamber with cylinder containing stranger mouse was regarded as chamber S1. Another side chamber with empty cylinder was regarded as chamber empty (chamber E). Then, the test mouse was placed into the center of the apparatus and allowed to freely explore for 5 min. Time spent in each chamber were recorded through Smart software (version 3.0; Panlab, S.L.U., Spain). Preference index for sociability was calculated as time spent in (chamber S1-chamber E)/total exploration time × 100%.

### Marble Burying Test

Repetitive behavior was examined using the marble burying test [39]. Each mouse was individually positioned in a standard mouse cage containing 4 cm of fresh bedding with 15 marbles arranged in a 3 × 5 grid on the top. Mice were allowed to freely explore for 20 min. The number of marbles buried were calculated.

### Open-field Test

Locomotion and anxiety-like behavior were analyzed using the open-field test [40]. The open-field test was conducted in an open chamber made of black Plexiglas (45 × 45 × 45 cm). Each mouse was individually placed into the center of the chamber and allowed to freely explore for 5 min. Total distance travelled and time spent in the center were recorded through Smart software.

### Elevated Plus Maze Test

Anxiety-like behavior was evaluated using the elevated plus maze test [41]. The elevated plus maze apparatus made of black Plexiglas was elevated 50 cm above the floor and consisted of two open arms (30 × 6 cm) alternating with two closed arms (30 × 6 with 25-cm high walls) and a common central platform (6 × 6 cm). Each mouse was positioned into the center of the maze and allowed to freely explore for 10 min. Time spent in closed arms and open arms were recorded through Smart software.

### Electrophysiological Recordings

Coronal slices containing hippocampus (400-μm thick) were prepared using vibratome (DTK-1000; Dosaka, Japan) and ice-cold artificial cerebrospinal fluid (aCSF) containing (in mM): NaCl 117, KCl 4.7, CaCl_2_ 2.5, MgCl_2_ 1.2, NaHCO_3_ 25, NaH_2_PO_4_ 1.2, and glucose 11 and bubbled with 95% O_2_ and 5% CO_2_. Brain slices were recovered at room temperature for at least 1 h, and transferred to a submerged recording chamber, continuously perfused with oxygenated aCSF at 30 ± 1 °C. A concentric bipolar stimulating electrode (FHC, Bowdoinham, ME, USA) was placed in the Schaeffer collateral fibers, and a glass microelectrode filled with 3 M NaCl solution was placed in the CA1 stratum radiatum to record field excitatory postsynaptic potential (fEPSP). Data were amplified and digitized using Axoclamp 2 A amplifier (Molecular Devices) and Digidata 1322 A (Molecular Devices), and analyzed using pClamp software (version 10.7; Molecular Devices). For long-term potentiation (LTP) and long-term depression (LTD) recordings, a stable 10–15 min baseline fEPSP response was recorded at an intensity that evoked fEPSPs at 30–40% of the maximum response. After baseline recordings, LTP was induced with high-frequency stimulation (HFS, three trains of stimuli, each 100 Hz for 1 s with 20 s inter-train interval). LTD was induced with low-frequency stimulation (LFS, 900 pulses at 1 Hz for 15 min).

### Golgi Staining

Golgi staining was performed using the sliceGolgi Kit (Bioenno sliceGolgi Kit; Bioenno tech) according to the manufacturer’s instructions. Mice were anesthetized and transcardially perfused with saline for 5 min followed by fixative solution for 20 min. Coronal slices containing hippocampus (150-μm thick) were prepared and immersed in impregnation solution for 9 days at room temperature in the dark. Brain slices were then treated with staining solution (8 min), post-staining solution (4 min), and mounted on gelatin-coated slides. Images of the neurons in the hippocampal CA1 region were acquired with Olympus BX63 microscope. Dendritic spines were counted in secondary dendrites of neurons on branches. Spine density was measured as the number of spines per 10 μm of the dendrite length. As described previously [42, 43], the protrusions of dendritic spines were divided into four subtypes containing mushroom (0.5–1.25 μm), thin (1.25–3.0 μm), filopodia (> 3 μm) and stubby (0.01–0.5 μm). The proportion and number of each spine subtypes were calculated. Sholl analysis was used to evaluate the dendritic arbors of each neuron as previously mentioned [44]. The number of basal or apical dendrite intersections at set distances from the soma was counted by a series of concentric rings with 10-μm intervals centered on the soma. All dendrites and spines within images were measured by investigators blinded to the experimental conditions with ImageJ software.

### Tissue Preparation and Immunoblotting

Whole-cell lysate and synaptoneurosome were prepared as described previously [39]. Hippocampus tissue was isolated from brain slices, and homogenized in iced-cold lysis buffer with protease and phosphatase inhibitor cocktail (Thermo Scientific, IL, USA). For whole-cell lysate, the homogenate was centrifuged (12,000 × *g*, 20 min), and the supernatant was prepared for immunoblotting. For synaptoneurosome, the homogenate was filtered sequentially through a 1-ml tuberculin syringe attached to a 13 mm diameter syringe filter holder (Millipore), a three-layer nylon (Tetko, 100-μm pore diameter), and a pre-wetted nitrocellulose filter (5 μm, Millipore). The final filtrate was spun briefly (1000 × *g*, 10 min), and the pellet was resuspended in the lysis buffer for immunoblotting.

Protein concentration was determined by Bio-Rad protein assay (Hercules, CA). Each sample of protein (10 μg) was separated by 10% SDS-PAGE, transferred to PVDF (Immobilon P membrane, Millipore), and blocked with 5% non-fat milk for 60 min. The membranes were incubated with primary antibodies overnight at 4 °C followed by the appropriate horseradish peroxidase-conjugated secondary antibodies at room temperature for 60 min. The primary antibodies used in the present study included anti-PSD95 antibody (1:5000; Cell signaling; #3409), anti-GluA2 antibody (1:5000; Millipore; MAB397), anti-AMPK antibody (1:2500; Cell signaling; #2532), anti-phospho-AMPK antibody (1:1000; Cell signaling; #2535), and anti-β-actin antibody (1:10,000; Abcam; ab6276). The blots were developed using ECL Plus detection reagent (PerkinElmer, Boston, MA, USA) and exposed to X-ray film (Fujifilm Super RX). Densitometric analysis of the immunoreactive bands was performed by Image J software.

### Quantitative Real-time PCR

Total RNA were isolated from the rodent hippocampal tissues by TRIzol RNA extraction kit (Invitrogen, Waltham, MA, USA) and processed according to the manufacturer’s instructions. RNA extract was then quantified and quality confirmed by using a Nanodrop spectrophotometer (260/280 and 260/230 ratios ≥ 1.8). Purified 0.5 μg RNA was reversed transcribed into cDNA by using M-MLV (Invitrogen, Waltham, MA, USA). Prepared DNA sample was then subjected to quantitative PCR analysis with SYBR Green (Bio-Rad, Hercules, CA, USA) and corresponding primers. Reactions were run in duplicated on a StepOnePlus RTPCR system (Applied Biosystems). Data were analyzed following the ΔΔCt method and expressed as fold control. β-actin was used as a normalization control. The following PCR primer sequences were used: PSD95, 5′-GACGCCAGCGACGAAGAG-3′ (Forward), 5′-CTCGACCCGCCGTTTG-3′ (Reverse); GluA2, 5′-ATCAAGAAGCCTCAGAAGTCCAAA-3′ (Forward), 5′-CTGACCCCAATGTAGGCAAAC-3′ (Reverse); β-actin, 5′-TACAACCTCCTTGCAGCTCC-3′ (Forward), 5′-ACAATGCCGTGTTCAATGG-3′ (Reverse).

### Statistical Analysis

All data were presents as mean ± SEM. Statistics were performed using GraphPad Prism 6 (San Diego, CA, USA). Experiments were analyzed using Student’s *t*-test, chi-square test, one-way ANOVA, two-way ANOVA, or two-way repeated measured (RM) ANOVA followed by Bonferroni *post-hoc* tests. Pearson correlation and linear regression analysis were applied for measuring the relationship between behavioral index and fEPSP slope. Error probabilities *p* < 0.05 were considered statistically significant.

## Results

### Effects of ENERGI on Autistic Behaviors in VPA-induced ASD Offspring

**Fig. 1:**
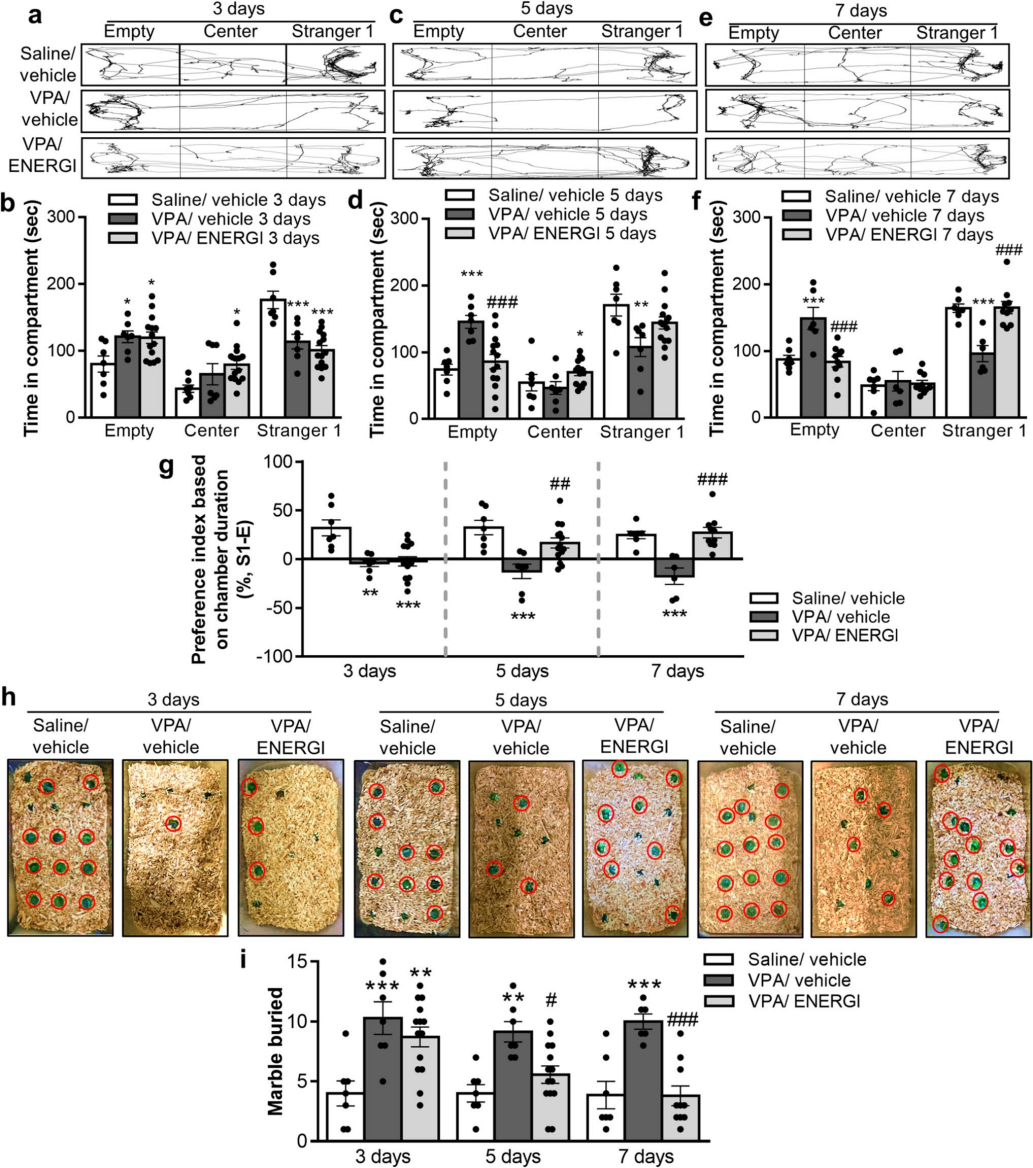
Effects of 3-, 5-, and 7-day ENERGI treatments on core autistic behaviors measured from VPA-induced offspring. (**a − b**) Representative traces (**a**) and time exploring three compartments (**b**) during the three-chamber sociability test after 3-day ENERGI or vehicle treatment in control and VPA-induced offspring. (**c − d**) Representative traces (**c**) and time exploring three compartments (**d**) during the three-chamber sociability test after 5-day ENERGI or vehicle treatment in control and VPA-induced offspring. (**e − f**) Representative traces (**e**) and time exploring three compartments (**f**) during the three-chamber sociability test after 7-day ENERGI or vehicle treatment in control and VPA-induced offspring. (**g**) Preference index calculated based on chamber duration in the three-chamber sociability test after 3-, 5-, and 7-day ENERGI or vehicle treatment in control and VPA-induced offspring. (**h − i**) Representative image at the end of the experiment (**h**) and the number of marbles buried (**i**) during the marble burying test after 3-, 5-, 7-day ENERGI or vehicle treatment in control and VPA-induced offspring. Mice number: Saline/vehicle 3 days = 7, VPA/vehicle 3 days = 7, VPA/ENERGI 3 days = 14, Saline/vehicle 5 days = 7, VPA/vehicle 5 days = 7, VPA/ENERGI 5 days = 14, Saline/vehicle 7 days = 7, VPA/vehicle 7 days = 6, VPA/ENERGI 7 days = 10. Data shown as mean ± SEM. *p < 0.05, **p < 0.01, ***p < 0.001 vs Saline/vehicle; ^*#*^*p* < 0.05, ^*##*^*p* < 0.01, ^*###*^*p* < 0.001 vs. VPA/vehicle using two-way ANOVA followed by Bonferroni *post-hoc* tests

To investigate the effect of ENERGI on the core behavioral features of ASD, we first assessed sociability using the three-chamber sociability test. The exploration time with Stranger 1 was shorter in vehicle-treated VPA-induced offspring than in vehicle-treated control offspring after 3-, 5-, and 7-day treatments. The exploration time with Stranger 1 gradually increased and reached a significant value on day 7 following ENERGI treatment in VPA-induced offspring compared with vehicle-treated VPA-induced offspring (area × group: 3-day, *F*_(4,75)_ = 11.89, *p* < 0.0001; 5-day, *F*_(4,75)_ = 9.40, *p* < 0.0001; 7-day, *F*_(4,60)_ = 14.59, *p* < 0.0001; Fig. 1a–f). Moreover, the sociability preference index revealed that 5-day and 7-day, but not 3-day, ENERGI treatments significantly improved the social defects in VPA-induced offspring (time, *F*_(2,71)_ = 0.2182, *p* = 0.8045; group, *F*_(2,71)_ = 27.13, *p* < 0.0001; time × group, *F*_(4,71)_ = 3.949, *p* = 0.0060; Fig. 1g). The marble burying test was employed to examine the effect of ENERGI on repetitive behavior. The number of marbles buried gradually decreased and reached a significant value on day 7 following ENERGI treatment in VPA-induced offspring compared with vehicle-treated VPA-induced offspring (time, *F*_(2,70)_ = 2.886, *p* = 0.0625; group, *F*_(2,70)_ = 24.23, *p* < 0.0001; time × group, *F*_(4,70)_ = 2.424, *p* = 0.0561; Fig. 1h–i). These results indicate that ENERGI treatment alleviated core behavioral deficits in VPA-induced ASD offspring.

**Fig. 2:**
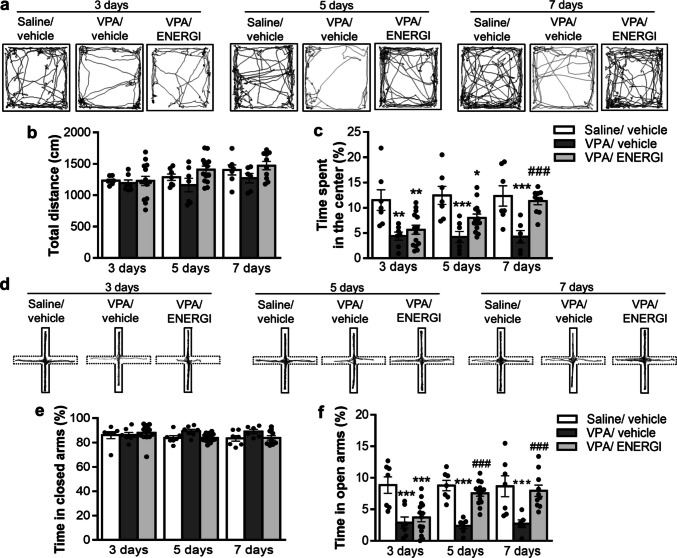
Effects of 3-, 5-, and 7-day ENERGI treatments on emotion dysregulations measured from VPA-induced offspring. (**a − c**) Representative traces (**a**), total distance (**b**), and time spent in the center of the open field (**c**) during the open-field test after 3-, 5-, and 7-day ENERGI or vehicle treatment in control and VPA-induced offspring. (**d − f**) Representative traces (**d**), and time spent in the closed arm (**e**) and open arm (**f**) during the elevated plus maze test after 3-, 5-, and 7-day ENERGI or vehicle treatment in control and VPA-induced offspring. Mice number: Saline/vehicle 3 days = 7, VPA/vehicle 3 days = 7, VPA/ENERGI 3 days = 14, Saline/vehicle 5 days = 7, VPA/vehicle 5 days = 7, VPA/ENERGI 5 days = 14, Saline/vehicle 7 days = 7, VPA/vehicle 7 days = 6, VPA/ENERGI 7 days = 10. Data shown as mean ± SEM. *p < 0.05, **p < 0.01, ***p < 0.001 vs Saline/vehicle; ^###^*p* < 0.001 vs. VPA/vehicle using two-way ANOVA followed by Bonferroni *post-hoc* tests

We also elucidated the effect of ENERGI on the emotional comorbidities of ASD. In the open-field test, no significant difference was observed in the total distance between the three groups after 3-, 5-, and 7-day treatments (time, *F*_(2,70)_ = 3.529, *p* = 0.0347; group, *F*_(2,70)_ = 3.706, *p* = 0.0295; time × group, *F*_(4,70)_ = 0.6985, *p* = 0.5956; Fig. 2a, b). However, the decreased time spent in the center gradually increased and reached statistical significance on day 7 following ENERGI treatment in VPA-induced offspring compared with vehicle-treated VPA-induced offspring (time, *F*_(2,70)_ = 2.080, *p* = 0.1326; group, *F*_(2,70)_ = 24.24, *p* < 0.0001; time × group, *F*_(4,70)_ = 1.795, *p* = 0.1395; Fig. 2c). While no significant difference was observed in the time spent in the closed arm among the three groups after 3-, 5-, and 7-day treatments in the elevated plus maze test (time, *F*_(2,70)_ = 0.4368, *p* = 0.6479; group, *F*_(2,70)_ = 2.686, *p* = 0.0752; time × group, *F*_(4,70)_ = 1.260, *p* = 0.2939; Fig. 2d, e), the reduced time spent in the open arm gradually increased and reached statistical significance on days 5 and 7 following ENERGI treatment in VPA-induced offspring compared with the vehicle-treated VPA-induced offspring (time, *F*_(2,70)_ = 1.726, *p* = 0.1856; group, *F*_(2,70)_ = 28.90, *p* < 0.0001; time × group, *F*_(4,70)_ = 3.074, *p* = 0.0216; Fig. 2f). These results indicate that ENERGI treatment rescued anxiety-like behaviors in the VPA-induced ASD offspring.

### Effects of ENERGI on Spine Morphology and Dendritic Arborization in the Hippocampus of VPA-induced ASD Offspring

**Fig. 3:**
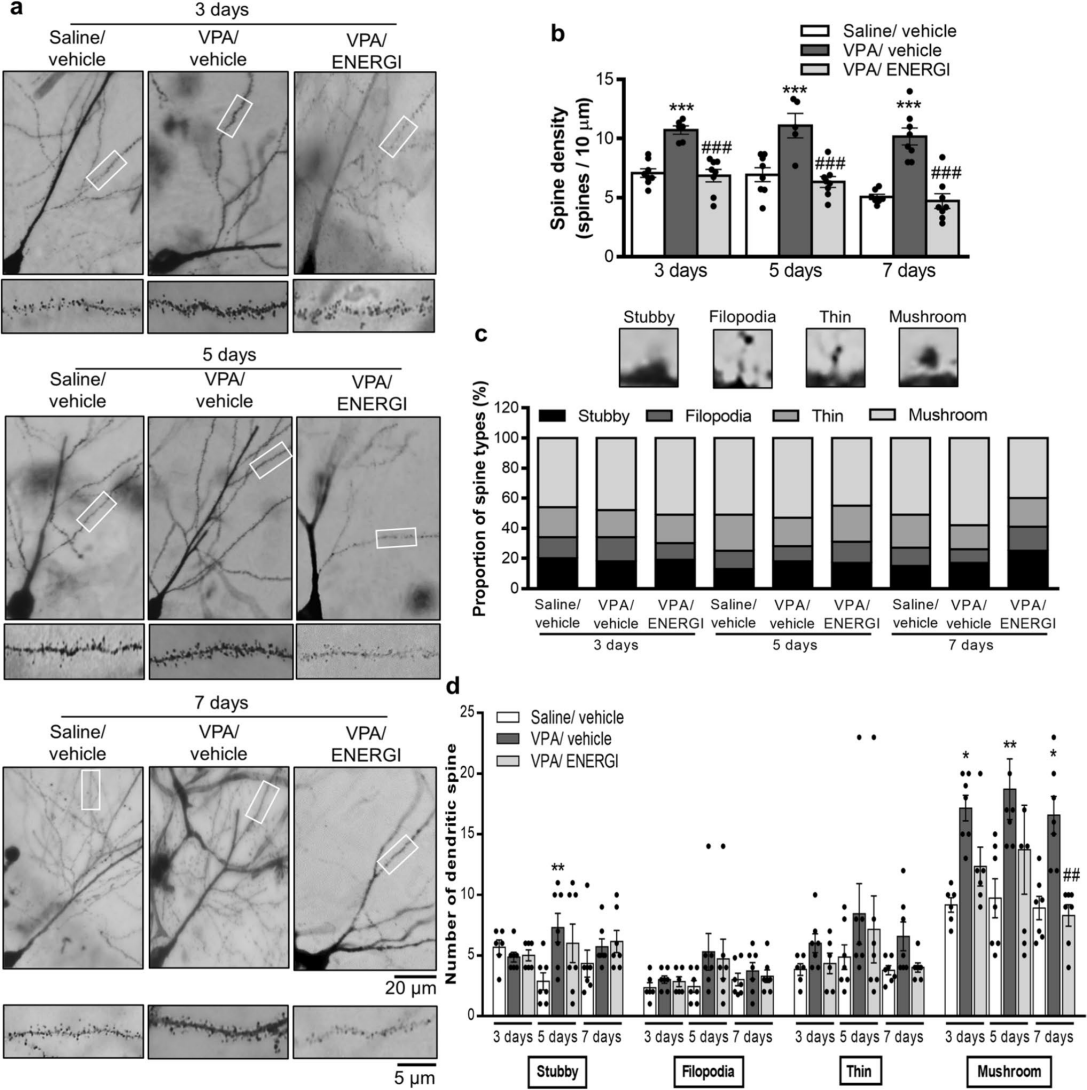
Effects of 3-, 5-, and 7-day ENERGI treatments on increased spine density and mushroom spine type calculated from the hippocampus of VPA-induced offspring. (**a − b**) Representative images (**a**) and summary bar graph (**b**) showing spine density measured from the secondary branch of apical dendrites of CA1 pyramidal neurons after 3-, 5-, and 7-day ENERGI or vehicle treatment in control and VPA-induced offspring. (**c − d**) Representative images and summary bar graphs showing the proportion of spine types (**c**) and the number of each dendritic spine types (**d**) measured from the CA1 pyramidal neurons after 3-, 5-, and 7-day ENERGI or vehicle treatment in control and VPA-induced offspring. For spine density, cells/mice number: Saline/vehicle 3 days = 8/4, VPA/vehicle 3 days = 6/4, VPA/ENERGI 3 days = 8/4, Saline/vehicle 5 days = 8/4, VPA/vehicle 5 days = 5/4, VPA/ENERGI 5 days = 8/3, Saline/vehicle 7 days = 7/4, VPA/vehicle 7 days = 8/4, VPA/ENERGI 7 days = 8/4. For spine types, cells/mice number: Saline/vehicle 3 days = 6/4, VPA/vehicle 3 days = 7/4, VPA/ENERGI 3 days = 6/4, Saline/vehicle 5 days = 7/4, VPA/vehicle 5 days = 7/4, VPA/ENERGI 5 days = 7/3, Saline/vehicle 7 days = 7/4, VPA/vehicle 7 days = 7/4, VPA/ENERGI 7 days = 7/4. Data shown as shown as mean ± SEM. *p < 0.05, **p < 0.01, ***p < 0.001 vs Saline/vehicle; ^##^*p* < 0.01, ^###^*p* < 0.001 vs. VPA/vehicle using two-way ANOVA followed by Bonferroni *post-hoc* tests

The hippocampus has been shown to contribute to the pathogenesis of ASD [45]. Aberrant development of the hippocampus, with larger volumes and abnormal functional connectivity, have been found in patients with ASD [45–48]. These alterations indicate both structural and functional impairments of the hippocampus in ASD. To determine the effects of ENERGI on the synaptic pathophysiology of ASD, we assessed dendritic spine density in the hippocampus of VPA-induced offspring following ENERGI treatments using Golgi staining. The increased spine density was significantly suppressed following 3-, 5-, and 7-day ENERGI treatment in the hippocampus of VPA-induced offspring compared with vehicle-treated VPA-induced offspring (time, *F*_(2,57)_ = 7.360, *p* = 0.0014; group, *F*_(2,57)_ = 57.69, *p* < 0.0001; time × group, *F*_(4,57)_ = 0.6396, *p* = 0.6364; Fig. 3a, b). We further categorized dendritic spines into stubby, filopodia-like, thin, and mushroom-like spines according to their morphologies. No significant difference was observed in the proportion of these spine categories among the hippocampal neurons (*χ*^*2*^ = 16.25, *p* = 0.8787; Fig. 3c), nor in the number of stubby, filopodia-like, or thin spines among three groups following 3-, 5-, and 7-day treatments. However, the number of mushroom-like spines was significantly higher in vehicle-treated VPA-induced offspring than in vehicle-treated control offspring, and was significantly ameliorated by ENERGI treatment on day 7 (stubby: time, *F*_(2,52)_ = 0.0485, *p* = 0.9527; group, *F*_(2,52)_ = 2.712, *p* = 0.0758; time × group, *F*_(4,52)_ = 2.094, *p* = 0.0948; filopodia: time, *F*_(2,52)_ = 1.937, *p* = 0.1544; group, *F*_(2,52)_ = 2.0442, *p* = 0.1397; time × group, *F*_(4,52)_ = 0.6204, *p* = 0.6500; thin: time, *F*_(2,52)_ = 2.019, *p* = 0.1431; group, *F*_(2,52)_ = 2.966, *p* = 0.0603; time × group, *F*_(4,52)_ = 0.1737, *p* = 0.9509; mushroom: time, *F*_(2,52)_ = 1.733, *p* = 0.1869; group, *F*_(2,52)_ = 15.60, *p* < 0.0001; time × group, *F*_(4,52)_ = 0.4994, *p* = 0.7363; Fig. 3d).

**Fig. 4:**
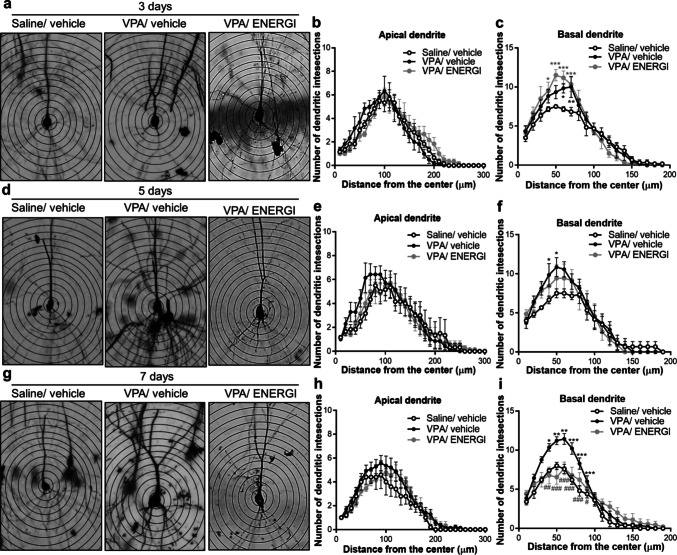
Effects of 3-, 5-, and 7-day ENERGI treatments on abnormal dendritic branch distribution observed from the hippocampus of VPA-induced offspring. (**a − c**) Representative images (**a**) and Sholl analyses of apical dendrites (**b**) and basal dendrites (**c**) from CA1 pyramidal neurons after 3-day ENERGI or vehicle treatment in control and VPA-induced offspring. (**d − f**) Representative images (**d**) and Sholl analyses of apical dendrites (**e**) and basal dendrites (**f**) from CA1 pyramidal neurons after 5-day ENERGI or vehicle treatment in control and VPA-induced offspring. (**g − i**) Representative images (**g**) and Sholl analyses of apical dendrites (**h**) and basal dendrites (**i**) from CA1 pyramidal neurons after 7-day ENERGI or vehicle treatment in control and VPA-induced offspring. Cells/mice number: Saline/vehicle 3 days = 6/4, VPA/vehicle 3 days = 6/4, VPA/ENERGI 3 days = 6/4, Saline/vehicle 5 days = 6/3, VPA/vehicle 5 days = 7/4, VPA/ENERGI 5 days = 7/3, Saline/vehicle 7 days = 5/4, VPA/vehicle 7 days = 14/4, VPA/ENERGI 7 days = 9/4. Data shown as mean ± SEM. *p < 0.05, **p < 0.01, ***p < 0.001 vs Saline/vehicle; ^#^*p* < 0.05, ^##^*p* < 0.01, ^###^*p* < 0.001 vs. VPA/vehicle using two-way RM ANOVA followed by Bonferroni *post-hoc* tests

Sholl’s analysis was used to assess the effect of ENERGI on dendritic arborization (Fig. 4). No significant differences were observed in the number of intersections between apical dendrites and Sholl rings from the hippocampal neurons among the three groups after 3-, 5-, and 7-day treatments (distance from soma × group: 3-day, *F*_(60,450)_ = 1.091, *p* = 0.3082; 5-day, *F*_(60,510)_ = 0.6841, *p* = 0.9655; 7-day, *F*_(60,750)_ = 0.7388, *p* = 0.9297; Fig. 4b, e, h). In contrast, the number of intersections between basal dendrites and Sholl rings from the hippocampal neurons was significantly higher in vehicle-treated VPA-induced offspring than in vehicle-treated control offspring. These abnormalities were significantly alleviated following 7-day ENERGI treatment (distance from soma × group: 3-day, *F*_(38,285)_ = 2.452, *p* < 0.0001; 5-day, *F*_(40,340)_ = 1.035, *p* = 0.4173; 7-day, *F*_(40,500)_ = 4.116, *p* < 0.0001; Fig. 4c, f, i). These results demonstrate that ENERGI treatment attenuated structural abnormalities in the synapses of the hippocampus in VPA-induced ASD offspring.

### Effects of ENERGI on Synaptic Plasticity in the Hippocampus of VPA-induced ASD Offspring

**Fig. 5:**
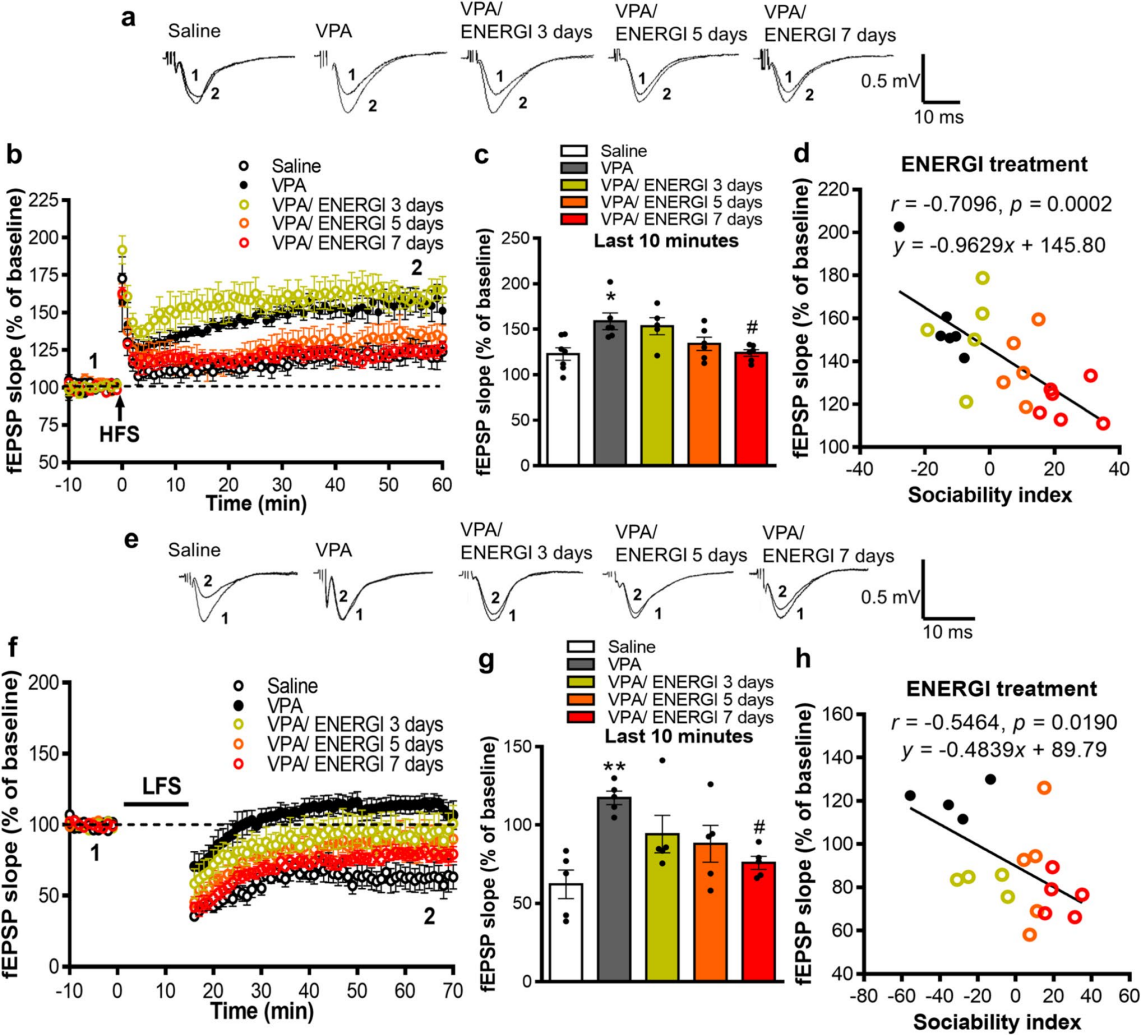
Effects of 3-, 5-, and 7-day ENERGI treatments on abnormal LTP and LTD recorded from the hippocampus of VPA-induced offspring. (**a − b**) Representative traces (**a**) and summary graph (**b**) of LTP in hippocampus after 3-, 5-, and 7-day ENERGI or vehicle treatment in control and VPA-induced offspring. (**c**) Averaged fEPSP slopes at 50 − 60 min after HFS. (**d**) Pearson correlation between fEPSP slope (50 − 60 min after HFS) and sociability index in the three-chamber sociability test. (**e − f**) Representative traces (**e**) and summary graph (**f**) of LTD in hippocampus after 3-, 5-, and 7-day ENERGI or vehicle treatment in control and VPA-induced offspring. (**g**) Averaged fEPSP slopes measured at 60 − 70 min after LFS. (**h**) Pearson correlation between fEPSP slope (60 − 70 min after LFS) and sociability index in the three-chamber sociability test. For LTP, slices/mice number: Saline = 5/3, VPA = 6/6, VPA/ENERGI 3 days = 6/5, VPA/ENERGI 5 days = 7/6, VPA/ENERGI 7 days = 6/5. For LTD, slices/mice number: Saline = 5/3, VPA = 5/4, VPA/ENERGI 3 days = 5/4, VPA/ENERGI 5 days = 5/5, VPA/ENERGI 7 days = 5/5. Data shown as mean ± SEM. *p < 0.05, **p < 0.01 vs Saline; ^#^*p* < 0.05 vs. VPA using one-way ANOVA followed by Bonferroni *post-hoc* tests

We also evaluated synaptic plasticity in the hippocampus of VPA-induced offspring following ENERGI treatments using electrophysiology. Hippocampal LTP was significantly enhanced in VPA-induced offspring compared with control offspring; this was gradually ameliorated and reached statistical significance on day 7 following ENERGI treatment in the VPA-induced offspring compared with naïve VPA-induced offspring (*F*_(4,25)_ = 5.057, *p* = 0.0040; Fig. 5a–c). Moreover, the sociability preference index was significantly correlated with LTP impairment following ENERGI treatment in VPA-induced offspring (*r* = −0.7096, *p* = 0.0002; Fig. 5d). Behavioral performance in the marble burying test and elevated plus maze test were also significantly correlated with LTP impairment (marble burying: *r* = 0.5699, *p* = 0.0056; elevated plus maze: *r* = −0.5868, *p* = 0.0041; Fig. S2a, c). No significant correlation was observed between performance in the open-field test and LTP impairment (*r* = −0.4059, *p* = 0.0609; Fig. S2b). Hippocampal LTD was impaired in VPA-induced offspring compared with control offspring, and was significantly mitigated by ENERGI treatment on day 7 in the VPA-induced offspring compared with the naïve VPA-induced offspring (*F*_(4,20)_ = 5.358, *p* = 0.0042; Fig. 5e–g). A significant correlation was observed between the sociability preference index and LTD abnormalities in VPA-induced offspring following ENERGI treatment (*r* = −0.5464, *p* = 0.0190; Fig. 5h). Additionally, behavioral performance in the open-field test and elevated plus maze test were significantly correlated with LTD abnormalities (open-field: *r* = −0.4972, *p* = 0.0358; elevated plus maze: *r* = −0.4935, *p* = 0.0034; Fig. S2e − f). No significant correlation was observed between performance in marble burying test and LTD abnormalities (*r* = 0.3752, *p* = 0.1250; Fig. S2d). These findings suggest that ENERGI exerts a protective effect on abnormal hippocampal synaptic plasticity in VPA-induced ASD offspring, which correlates with improvements in ASD-related behaviors, especially sociability.

### Effects of ENERGI on Autistic Behaviors in the VPA-induced ASD Offspring Were Similar to DCS

**Fig. 6:**
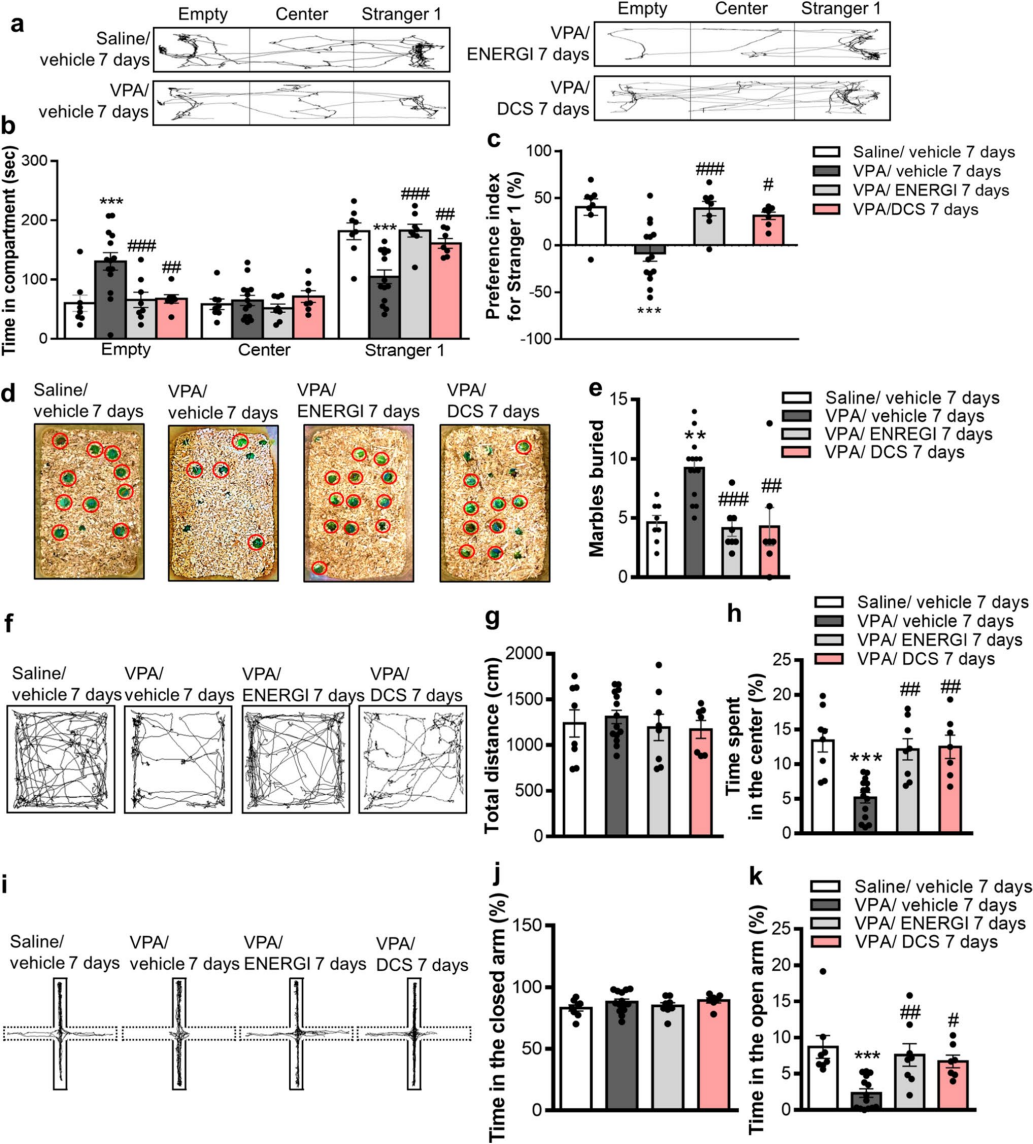
Comparisons of the effect of ENERGI and DCS treatments on ASD-related behaviors in VPA-induced offspring. (**a − b**) Representative traces (**a**) and time exploring three compartments (**b**) during the three-chamber sociability test after ENERGI, DCS, or vehicle treatment in control and VPA-induced offspring. (**c**) Preference index calculated based on chamber duration in the three-chamber sociability test after ENERGI, DCS, or vehicle treatment in control and VPA-induced offspring. (**d − e**) Representative image at the end of the experiment (**d**) and the number of marbles buried (**e**) during the marble burying test after ENERGI, DCS, or vehicle treatment in control and VPA-induced offspring. (**f − h**) Representative traces (**f**), total distance (**g**), and time spent in the center of the open field (**h**) during the open-field test after ENERGI, DCS, or vehicle treatment in control and VPA-induced offspring. (**i − k**) Representative traces (**i**), and time spent in the closed arm (**j**) and open arm (**k**) during the elevated plus maze test after ENERGI, DCS, or vehicle treatment in control and VPA-induced offspring. Mice number: Saline/vehicle = 8, VPA/vehicle = 14, VPA/ENERGI = 8, VPA/DCS = 7. Data shown as mean ± SEM. **p < 0.01, ***p < 0.001 vs Saline/vehicle; ^#^*p* < 0.05, ^##^*p* < 0.01, ^###^*p* < 0.001 vs. VPA/vehicle using one-way ANOVA or two-way ANOVA followed by Bonferroni *post-hoc* tests

We subsequently investigated whether the effects of ENERGI on ASD were comparable to the available treatments for ASD using DCS. We examined the autistic behavior of VPA-induced ASD offspring to compare the effects of 7-day ENERGI and DCS treatments. Notably, the exploration time with Stranger 1 was significantly rescued after 7-day ENERGI and DCS treatments in VPA-induced offspring compared with vehicle-treated VPA-induced offspring (area × group, *F*_(6,99)_ = 10.77, *p* < 0.0001; Fig. 6a, b). The sociability preference index was also significantly reversed following both treatments in VPA-induced offspring (*F*_(3,33)_ = 9.630, *p* = 0.0001; Fig. 6c). Additionally, the number of marbles buried in the marble burying test were increased following both treatments in VPA-induced offspring (*F*_(3,33)_ = 9.552, *p* = 0.0001; Fig. 6d, e). No significant effect was observed in the total distance between the four groups in the open field test (*F*_(3,33)_ = 0.3464, *p* = 0.7919; Fig. 6f, g). The time spent in the center was significantly ameliorated following both treatments in VPA-induced offspring (*F*_(3,33)_ = 10.88, *p* < 0.0001; Fig. 6h). No significant difference was observed in the time spent in the closed arm of the elevated plus maze among the four groups (*F*_(3,33)_ = 1.185, *p* = 0.3303; Fig. 6i, j). The time spent in the open arm was increased following both treatments in the VPA-induced offspring (*F*_(3,33)_ = 7.969, *p* = 0.0004; Fig. 6k). These results indicate that ENERGI treatment rescued ASD-like behaviors in the VPA-induced ASD offspring, equivalent to DCS treatment.

### ENERGI Demonstrated Superior Effects on Synaptic Structure Abnormalities in VPA-induced ASD Offspring to DCS

**Fig. 7:**
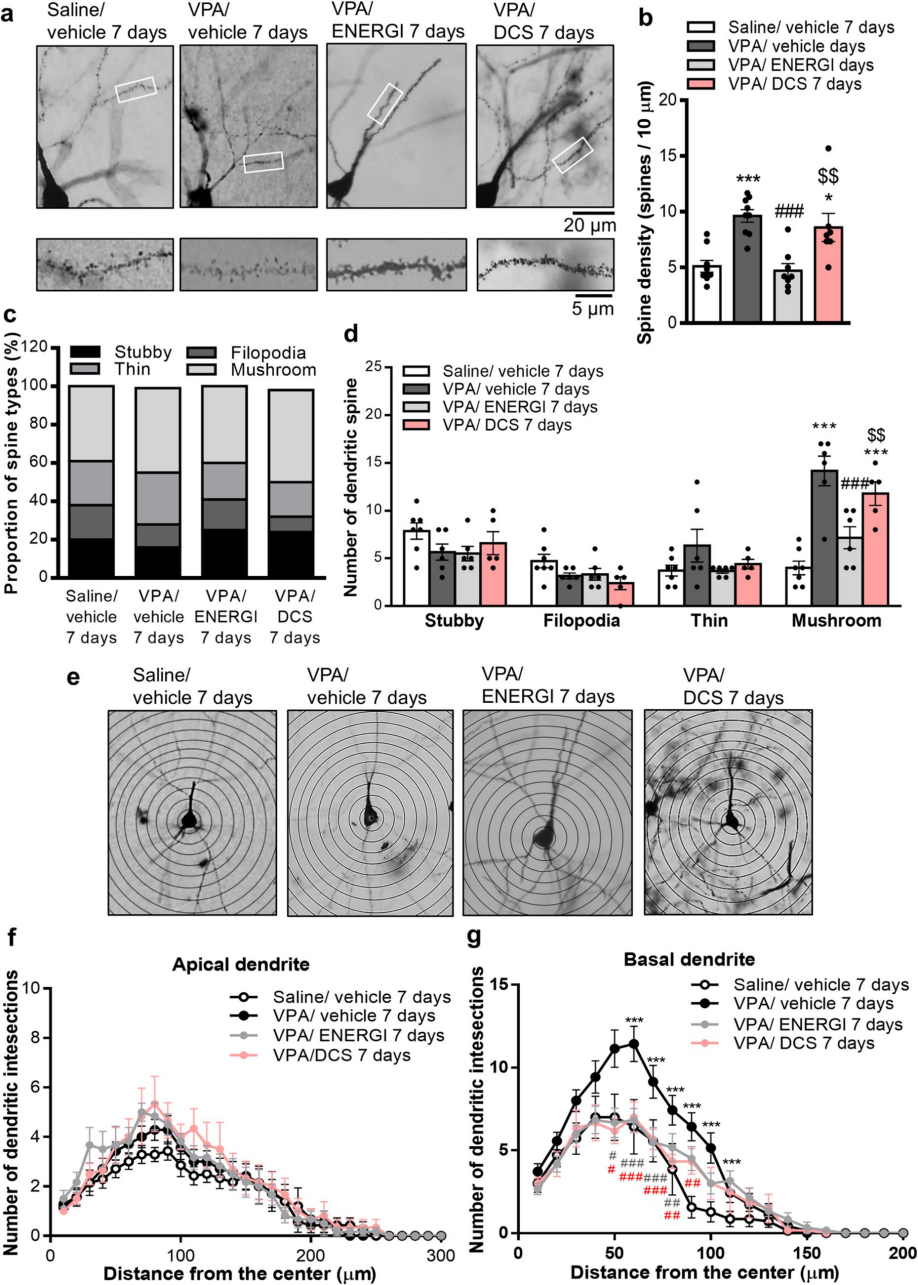
Comparisons of the effect of ENERGI and DCS treatments on spine density, spine type, and dendritic branch distribution in the hippocampus of VPA-induced offspring. (**a − b**) Representative images (**a**) and summary bar graph (**b**) showing spine density measured from the secondary branch of apical dendrites of CA1 pyramidal neurons after ENERGI, DCS, or vehicle treatment in control and VPA-induced offspring. (**c − d**) Representative images and summary bar graphs showing the proportion of spine types (**c**) and the number of each dendritic spine types (**d**) measured from the CA1 pyramidal neurons after ENERGI, DCS, or vehicle treatment in control and VPA-induced offspring. (**e − g**) Representative images (**e**) and Sholl analyses of apical dendrites (**f**) and basal dendrites (**g**) from CA1 pyramidal neurons after ENERGI, DCS, or vehicle treatment in control and VPA-induced offspring. For spine density, cells/mice number: Saline/vehicle = 9/4, VPA/vehicle = 9/5, VPA/ENERGI = 8/4, VPA/DCS = 7/4. For spine types, cells/mice number: Saline/vehicle = 7/4, VPA/vehicle = 6/5, VPA/ENERGI = 6/4, VPA/DCS = 5/4. For dendritic branch distribution, cells/mice number: Saline/vehicle = 7/4, VPA/vehicle = 7/5, VPA/ENERGI = 6/4, VPA/DCS = 6/4. Data shown as mean ± SEM. *p < 0.05, ***p < 0.001 vs Saline/vehicle; ^#^*p* < 0.05, ^##^*p* < 0.01, ^###^*p* < 0.001 vs. VPA/vehicle; ^*$$*^*p* < 0.01 vs. VPA/ENERGI using one-way ANOVA, two-way ANOVA, or two-way RM ANOVA followed by Bonferroni *post-hoc* tests

We compared the effect of 7-day ENERGI and DCS treatments on spine morphology and dendritic arborization in VPA-induced ASD offspring. Notably, 7-day ENERGI treatment, but not 7-day DCS treatment, significantly reversed the increased spine density in the hippocampus of VPA-induced offspring (*F*_(3,29)_ = 11.23, *p* < 0.0001; Fig. 7a, b). No significant difference was observed in the proportions of the spine categories among the hippocampal neurons (*χ*^*2*^ = 10.28, *p* = 0.3282; Fig. 7c), nor in the number of stubby, filopodia-like, or thin spines among the four groups. The elevated number of mushroom-like spines was significantly alleviated after 7-day ENERGI treatment, but not after 7-day DCS treatment (spine type × group: *F*_(9,80)_ = 7.814, *p* < 0.0001; spine type: *F*_(3,80)_ = 29.81, *p* < 0.0001; group: *F*_(3,80)_ = 6.023, *p* = 0.0009; Fig. 7d). Furthermore, Sholl analysis revealed no significant differences in the number of intersections between apical dendrites and Sholl rings from hippocampal neurons among the four groups (distance from soma, *F*_(30,660)_ = 57.21, *p* < 0.0001; group,* F*_(3,22)_ = 1.664, *p* = 0.2038; distance from soma × group, *F*_(90,660)_ = 0.7369, *p* = 0.9645; Fig. 7e, f). However, both 7-day ENERGI and DCS treatments significantly suppressed the increased number of intersections between basal dendrites and Sholl rings from the hippocampal neurons in VPA-induced offspring compared with vehicle-treated VPA-induced offspring (distance from soma, *F*_(20,440)_ = 88.75, *p* < 0.0001; group,* F*_(3,22)_ = 5.552, *p* = 0.0054; distance from soma × group,* F*_(60,440)_ = 2.313, *p* < 0.0001; Fig. 7g). These results demonstrated the superior effects of ENERGI treatment on synaptic structural anomalies in the hippocampus of VPA-induced ASD offspring.

### Effects of ENERGI on Synaptic Plasticity in the VPA-induced ASD Offspring Were Similar to DCS

**Fig. 8:**
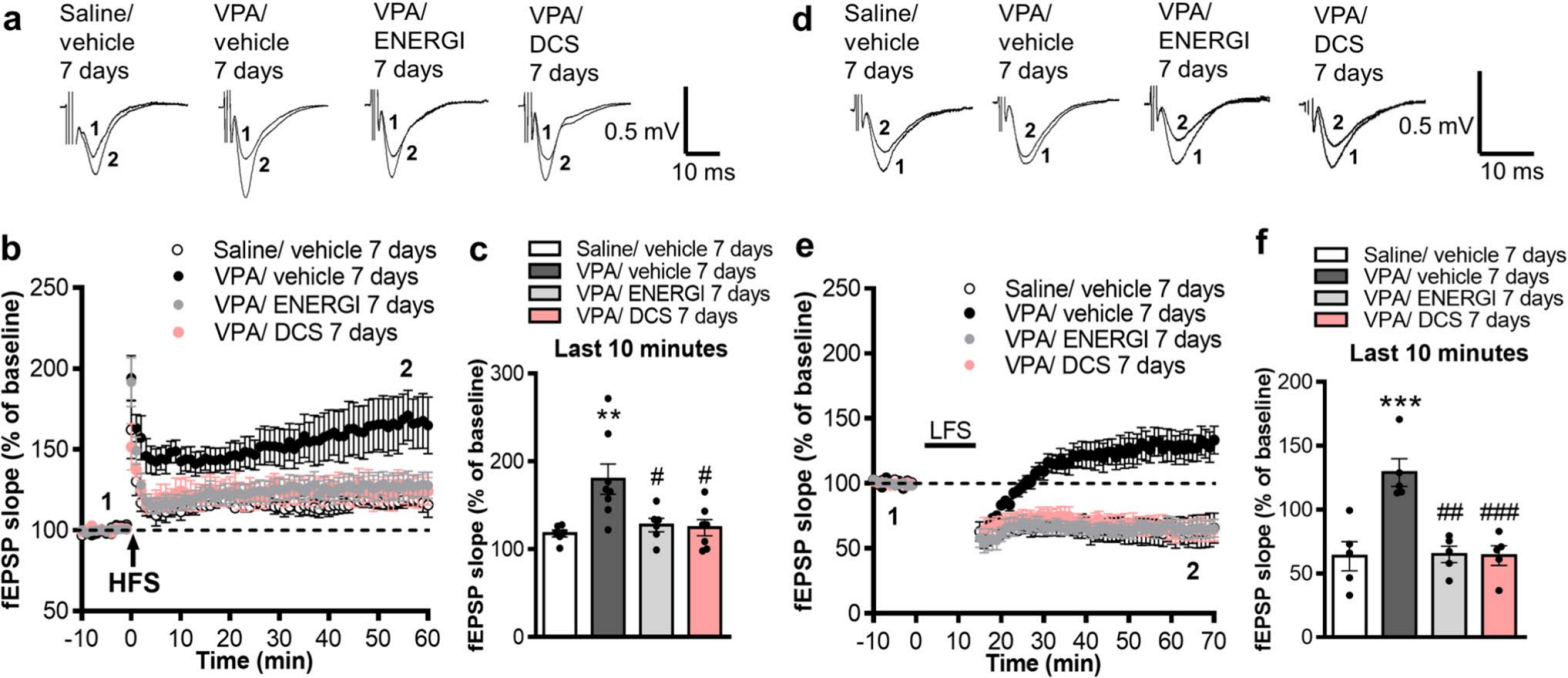
Comparisons of the effect of ENERGI and DCS treatments on LTP and LTD in the hippocampus of VPA-induced offspring. (**a − b**) Representative traces (**a**) and summary graph (**b**) of LTP in hippocampus after ENERGI, DCS, or vehicle treatment in control and VPA-induced offspring. (**c**) Averaged fEPSP slopes measured at 50 − 60 min after HFS. (**d − e**) Representative traces (**d**) and summary graph (**e**) of LTD in hippocampus after ENERGI, DCS, or vehicle treatment in control and VPA-induced offspring. (**f**) Averaged fEPSP slopes measured at 50 $-$ 60 min after LFS. For LTP, slices/mice number: Saline/vehicle = 6/3, VPA/vehicle = 8/4, VPA/ENERGI = 6/3, VPA/DCS = 7/3. For LTD, slices/mice number: Saline/vehicle = 5/3, VPA/vehicle = 5/4, VPA/ENERGI = 5/3, VPA/DCS = 5/3. Data shown as mean ± SEM. **p < 0.01, ***p < 0.001 vs Saline/vehicle; ^#^*p* < 0.05, ^##^*p* < 0.01, ^###^*p* < 0.001 vs. VPA/vehicle using one-way ANOVA followed by Bonferroni *post-hoc* tests

We compared the effect of 7-day ENERGI and DCS treatments on synaptic plasticity in VPA-induced ASD offspring. Both treatments significantly relieved the enhanced LTP (*F*_(3,23)_ = 6.290, *p* = 0.0028; Fig. 8a–c) and LTD (*F*_(3,16)_ = 12.16, *p* = 0.0002; Fig. 8d–f) in the hippocampus of VPA-induced offspring compared with vehicle-treated VPA-induced offspring. These results indicate that ENERGI treatment exerts protective effects equivalent to that of DCS against synaptic plasticity impairments in VPA-induced ASD offspring.

### Effects of ENERGI on AMPK and Synapse-associated Proteins in VPA-induced ASD Offspring

**Fig. 9:**
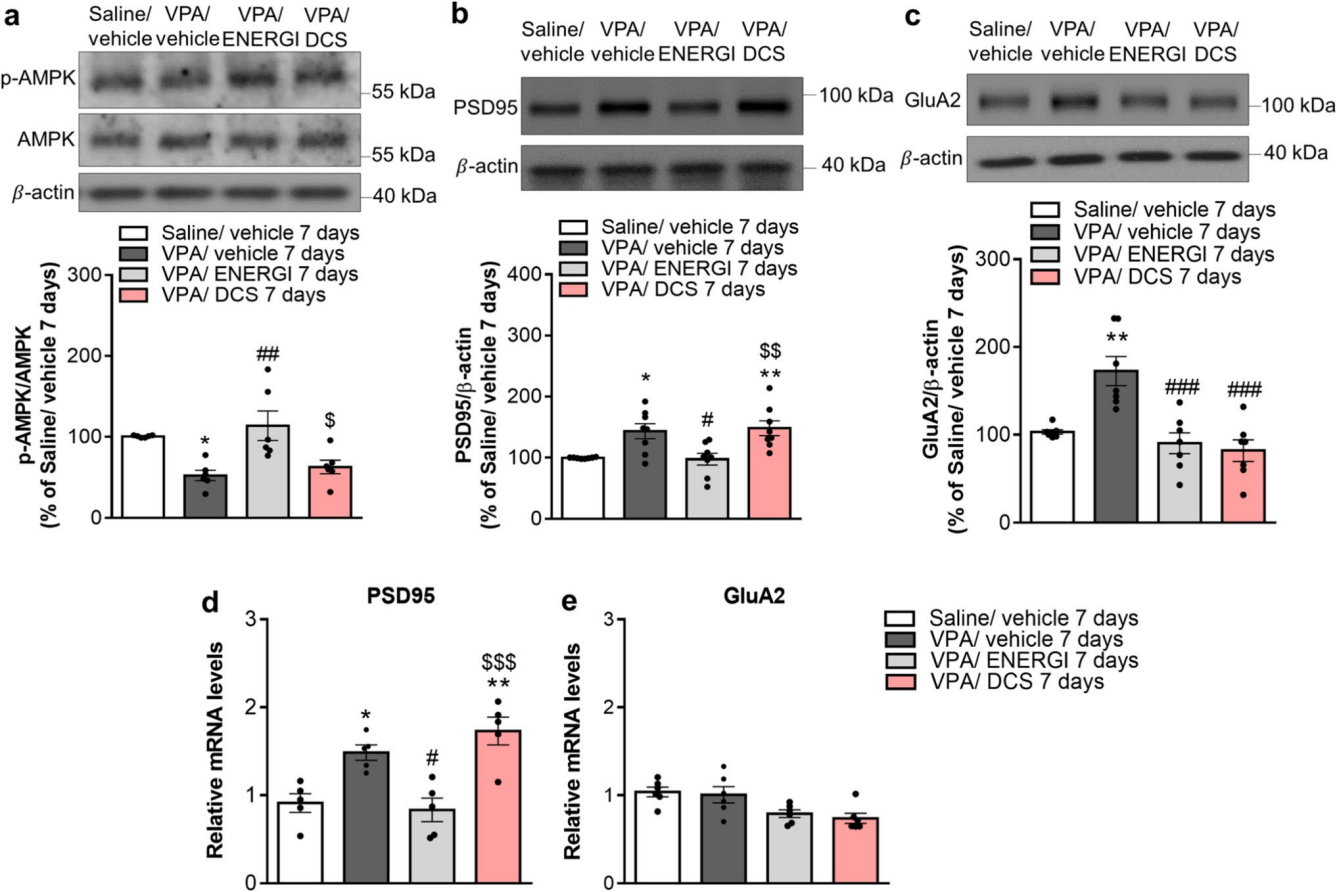
Effects of ENERGI on AMPK and synapse-associated proteins in VPA-induced offspring. (**a − c**) Representative immunoblot and summary bar graph showing the phosphorylation levels of AMPK (**a**), levels of PSD95 (**b**), and synaptic levels of GluA2 (**c**) in the hippocampus after 7-day ENERGI, DCS, or vehicle treatment in control and VPA-induced offspring. (**d − e**) Summary bar graph showing the mRNA levels of PSD95 (**d**), and GluA2 (**e**) in the hippocampus after 7-day ENERGI, DCS, or vehicle treatment in control and VPA-induced offspring. For protein levels, p-AMPK, mice number = 6 in each group; PSD95, mice number = 8 in each group; GluA2, mice number = 7 in each group. For mRNA levels, PSD95, mice number = 5 in each group; GluA2, mice number = 6 in each group. Data shown as mean ± SEM. *p < 0.05, **p < 0.01 vs Saline/vehicle; ^#^*p* < 0.05, ^##^*p* < 0.01, ^###^*p* < 0.001 vs. VPA/vehicle; ^*$*^*p* < 0.05, ^*$$*^*p* < 0.01, ^*$$$*^*p* < 0.001 vs. VPA/ENERGI using one-way ANOVA followed by Bonferroni *post-hoc* tests

We compared the molecular targets underlying the beneficial effects of ENERGI. Western blot analyses of whole-cell lysate revealed reduced AMPK phosphorylation and increased PSD95 protein levels in the hippocampus of vehicle-treated VPA-induced offspring compared with vehicle-treated control offspring, which were significantly rescued following 7-day ENERGI treatment, but not 7-day DCS treatment (p-AMPK,* F*_(3,20)_ = 7.784, *p* = 0.0012; PSD95, *F*_(3,28)_ = 7.696, *p* = 0.0007; Fig. 9a, b). In synaptoneurosomal tissue, enhanced GluA2 protein levels in the hippocampus of VPA-induced offspring were significantly ameliorated following 7-day ENERGI and DCS treatments (*F*_(3,24)_ = 11.87, *p* < 0.0001; Fig. 9c). The enhanced mRNA levels of PSD95 in the hippocampus of vehicle-treated VPA-induced offspring were significantly reversed following 7-day ENERGI treatment, but not 7-day DCS treatment (*F*_(3,16)_ = 12.48, *p* = 0.0002; Fig. 9d). No significant difference was found in the mRNA levels of GluA2 in the hippocampus among all groups (*F*_(3,20)_ = 5.433, *p* = 0.0067; Fig. 9e). These results indicate that the therapeutic effects of ENERGI may be mediated by increasing AMPK phosphorylation and the regulation of synapse-associated proteins in the hippocampus of VPA-induced ASD offspring.

## Discussion

This study provides evidence that ENERGI exerts profound protection against core autistic behaviors and emotional comorbidities in an ASD animal model. Importantly, we highlight a pivotal role of AMPK activation in the regulation of synaptic morphological and functional anomalies in ASD pathogenesis.

Pharmacological medications that can completely ameliorate core ASD symptoms remain unavailable. Psychotropic drugs used for treating ASD mainly address associated symptoms with limited efficacy on core symptoms [6]. Moreover, antipsychotic drugs often cause adverse effects, including sedation, extrapyramidal symptoms, and weight gain [49]. In this study, we assessed the effect of oral ENERGI treatment at three time points (on days 3, 5, and 7). ENERGI treatment for 7 days exerted no significant change in control offspring (Fig. S3 and Fig. S4). By contrast, ENERGI treatment relieved core ASD-like behaviors, social deficits and repetitive behaviors, in a time-dependent manner, with significant effects on day 7 in VPA-induced ASD offspring. Consistently, 7-day ENERGI treatment gradually restored synaptic structural and functional impairments in the hippocampus of VPA-induced offspring. Importantly, daily ENERGI administration for 7 days did not alter baseline locomotor activity (Fig. 2a–b) or body weight (Fig. S1a–c). These findings support the potential of ENERGI as an effective therapeutic candidate for ASD with fewer side effects.

Early intervention for ASD demonstrates greater efficacy and improved long-term outcomes [50, 51]. In line with clinical studies, early postnatal treatment markedly rescued autistic behaviors in adult mice with ASD [52, 53]. The typical age at ASD diagnosis is approximately 30–120 months [54, 55]. Accordingly, we applied ENERGI treatment to VPA-induced mice from PND28, which corresponds to approximately 10 years of age in humans [56, 57]. ENERGI significantly improved core ASD symptoms after a 7-day treatment, with effects persisting for at least 7 days post-treatment (Fig. S5). These findings suggest that ENERGI confers both normalizing and lasting benefits in our ASD model, highlighting its therapeutic potential for ASD. Future studies should determine the duration of these therapeutic effects and evaluate the safety of long-term administration.

This study revealed novel aspects of neuronal morphology and synaptic plasticity anomalies in the hippocampus of the VPA model. Hippocampal pyramidal neurons exhibit two distinct arbors: basal dendrites, emerging from the base of soma, receive local CA3 inputs for rapid synaptic integration and plasticity; and apical dendrites, extending far from soma, integrate distal inputs from other brain areas for higher-order processing [58, 59]. We only observed increased arborization in basal dendrites in the CA1 region of VPA-induced animals. Since different ASD susceptibility genes selectively affect basal or apical dendrites [60–62], these findings suggest dendritic compartment-specific regulation in the hippocampal CA1 neurons of VPA-induced animals. We also observed elevated spine density and more mushroom-like spines in the hippocampus of the VPA model. Similarly, previous studies reported increased pre- and postsynaptic markers at 4 − 5 week of age in VPA-induced offspring [63]. By contrast, hippocampal spine loss has been reported at 8 − 9 weeks of age in the VPA model [64, 65]. Decreased pruning markers have been linked to increased spine density and postsynaptic markers from childhood to adolescence in postmortem temporal lobes of individuals with ASD [66]. Additionally, the VPA model showed enhanced embryonic but reduced adult hippocampal neurogenesis [67]. Together, these findings suggest that increased spine density at 4 − 5 weeks may result from reduced pruning, while decreased spine density at 8 − 9 week of age may reflect impaired neurogenesis. Future studies are needed to clarify the age-dependent profile of these synaptic changes in ASD. Alterations in synaptic plasticity across multiple brain regions, including the hippocampus, neocortex, prefrontal cortex, and amygdala, have been implicated in VPA-induced ASD pathology [68–71]. Notably, distinct LTP patterns have been reported in different neural circuits. For instance, previous studies in VPA-exposed rats have demonstrated attenuated LTP at perforant pathway-dentate gyrus synapses [71], whereas our findings reveal enhanced LTP in the Schaffer collateral-CA1 pathway of mice. These findings underscore the circuit-specific nature of plasticity changes and support the role of synaptic dysregulation in ASD-like behaviors.

Abnormal serum AMPK levels have been measured in patients with ASD [72]. Alterations in protein expression or activity of AMPK have been reported in the brain of ASD animals, most of which are linked to metabolic stress or mitochondrial dysfunction in ASD [73–76]. Moreover, genetic or pharmacological modulations have been found to alleviate ASD-related behaviors in animals through the correction of AMPK activity [35, 76–78], highlighting the importance of targeting AMPK in the treatment of ASD. Our study shows that ENERGI profoundly ameliorates behavioral deficits in ASD animals by enhancing AMPK phosphorylation. These findings offer novel insights that ENERGI, a purine analogue, addresses ASD symptoms through AMPK activation.

This is the first study to demonstrate that normalizing AMPK activity by ENERGI rescues impaired synaptic plasticity and dendritic structure in an ASD model. AMPK plays an important role as a cellular energy sensor that regulates ATP homeostasis [79]. Under metabolic stress, excessive AMPK activation suppresses axonal and dendritic growth during neurodevelopment [26]. AMPK hyperactivation, triggered by an increased cellular AMP/ATP ratio under metabolic stress, has been linked to LTP impairment [25]. ENERGI is a purine analogue structurally similar to adenine and has been shown to activate AMPK and increase cellular ATP levels [31, 80, 81]. Notably, AMPK maintains cellular metabolic homeostasis in coordination with the mammalian target of rapamycin (mTOR) [82, 83]. Moreover, AMPK regulation on synaptic plasticity and integrity has been linked to mTOR-dependent protein translation [25, 29]. Our previous study further identified the AMPK–mTOR pathway as a potential therapeutic target for synaptic dysfunction in VPA-induced ASD animals [35]. Accordingly, these findings suggest that ENERGI may restore synaptic function and structure in ASD offspring by normalizing AMPK activity within this metabolic homeostasis network involving mTOR.

Considering PSD95 as a scaffold protein localized at the glutamatergic synapse essential for the synaptic plasticity and dendritic spine morphogenesis [84, 85], the present study explored its potential involvement in the effect of ENERGI treatment on ASD. We found that in parallel with the effect of ENERGI on AMPK phosphorylation, increased PSD95 protein levels in VPA-induced offspring were rescued from day 3 of ENERGI treatment (Fig. S6 and Fig. 9). Elevated spine density in the VPA-induced offspring was also rescued from day 3 of ENERGI treatment. Notably, PSD95 is one of the synaptic proteins modulated by mTOR-dependent protein translation [86–88]. mTOR plays a critical role in regulating synaptic morphology in autism [66, 89]. Evidence has shown that AMPK ameliorates dendritic pathology, including PSD95 expression, through the mTOR pathway [90]. These findings suggest that PSD95 participates in the restorative effects of ENERGI on synaptic structure through the AMPK–mTOR axis. Increased synaptic α-amino-3-hydroxy-5-methyl-4-isoxazolepropionic acid (AMPA) receptor subunit GluA2 levels in VPA-induced offspring were not reversed until day 7 of ENERGI treatment (Fig. S6 and Fig. 9). Moreover, the changes in GluA2 were found in the synaptic fraction, not mRNA level, of the hippocampus of VPA-induced offspring (Fig. 9). Studies have found that PSD95 directly interacts with auxiliary proteins of AMPA receptors, which are necessary for the recruitment and stabilization of AMPA receptors at the postsynaptic region [91, 92]. Overexpression of PSD95 selectively enhanced AMPA receptor-mediated synaptic response, and knockout of PSD95 reduced the AMPA receptor-mediated synaptic response [93, 94]. Increased PSD95 recruits more AMPA receptors to the synaptic region during activity-dependent trafficking, rather than constitutive trafficking [93, 95]. Together, our observations suggest that the therapeutic effects of ENERGI, an AMPK activator, on VPA-induced offspring may begin with ameliorating PSD95 levels, then normalizing synaptic AMPA receptors, and ultimately restoring synaptic plasticity.

Glutamatergic dysfunction is well documented in the pathogenesis of ASD [96]. Interventions involving glutamatergic modulation for neuropsychiatric disorders have demonstrated promising therapeutic effects [97]. Leveraging DCS as an NMDA receptor partial agonist, its curative effects on behavioral dysfunctions in ASD have been investigated in animal and clinical studies [98–102]. Here, we used DCS as a reference drug and compared the effects of ENERGI and DCS in the VPA-induced ASD model. ENERGI treatment for 7 days exerted effects similar to DCS on ASD-related behaviors and impaired NMDA receptor-dependent LTP and LTD in VPA-induced offspring. Furthermore, we found that ENERGI and DCS treatments suppressed the elevated synaptic GluA2 levels in the hippocampus of VPA-induced offspring. Considering our previous findings that DCS ameliorates ASD-like deficits through facilitating synaptic GluA2-containing AMPA receptor removal in VPA-induced offspring [38]. These results suggest that the ameliorating AMPA receptor defects and targeting the glutamatergic dysfunction contribute to the beneficial effects of ENERGI on behavioral and synaptic deficits in animals with ASD.

Notably, superior modulation of spine density and spine type alterations in the hippocampus of VPA-induced ASD offspring were demonstrated by 7-day ENERGI treatment than DCS treatment. Combining the current results with our previous findings in the amygdala, where the same DCS treatment significantly suppressed increased spine density [38]. These results suggest a brain region-specific effect of DCS in VPA-induced offspring. In parallel, the lack of AMPK phosphorylation and elevation of PSD95 protein and mRNA levels in the hippocampus were reversed by 7-day ENERGI treatment, but not DCS treatment. These distinct mechanistic profiles of ENERGI and DCS may arise from their different molecular targets. While DCS, an NMDA receptor partial agonist, directly acts at the glutamatergic synapse [103], ENERGI, a purine analogue, may exert its effects through metabolic regulation [31, 80, 81]. By activating AMPK, ENERGI could engage downstream signaling, such as the mTOR pathway, thereby modulating synaptic proteins, including PSD95, that are crucial for synaptic integrity and morphology [29, 87, 90]. This may underlie ENERGI’s superior efficacy in normalizing hippocampal dendritic spine density and spine type alterations, highlighting its unique mechanism beyond conventional glutamatergic modulation by DCS.

While this study provides compelling evidence for the therapeutic effects of ENERGI on VPA-induced ASD offspring, several limitations should be noted. First, we focused on male offspring, as ASD is a male-biased disorder [4], and autistic behaviors are more pronounced in VPA-induced male offspring [63, 104, 105]. However, distinct pathological alterations in the brain have been reported between male and female offspring of the VPA model [63, 106–108]. Future investigations in female offspring are warranted to delineate sex-specific mechanisms and therapeutic responses. Second, mice with insufficient ENERGI intake (< 80% of the target dose) were excluded from the present study based on predefined criteria. Although these exclusions did not significantly affect the statistical outcomes or interpretation of results, the relatively small sample size may limit the generalizability of our findings to broader populations. Third, behavioral tests were conducted with 4-h intervals to fit our treatment timeline. Baseline locomotor activity (Fig. 2a–b) did not differ across groups. Other behavioral outcomes were consistent with prior studies using similar paradigms in the VPA model [109]. These results support the reliability of present measurements. Nevertheless, whether shorter inter-test intervals influence the behavioral response to ENERGI warrants further investigation. Future studies with longer intervals could further confirm the independence of drug responsiveness in this model. Besides, although the present study strongly correlates ENERGI’s effects with AMPK activation, causal evidence remains to be established. Previous studies have found that AMPK inhibitor heightened hippocampal LTP under control conditions [25] and altered behavioral performance in healthy animals [110]. Given that ENERGI was administered over an extended period in this study, cotreatment with an AMPK inhibitor was not feasible due to potential toxicity concerns. Future investigations using brain- or hippocampus-specific AMPK knockout models are warranted to clarify the mechanistic contribution of AMPK activation to ENERGI’s actions. Finally, we primarily focused on the synaptic structure and function in the hippocampus. Given that ASD pathology involves whole-brain networks [111], future studies on other ASD-relevant brain regions, including the prefrontal cortex and amygdala, are required.

In conclusion, this study demonstrated the significant therapeutic effects of ENERGI on behavioral deficits and synaptic alterations in an ASD animal model. The improvement in sociability and reduction of repetitive behaviors provide preclinical evidence supporting ENERGI as a potential treatment for core ASD symptoms. Moreover, the restoration of synaptic plasticity and dendritic spine morphology further highlights ENERGI’s efficacy in targeting the synaptic pathophysiology of ASD. Mechanistically, our findings suggest that ENERGI exerts its therapeutic effects through AMPK activation, accompanied by the normalization of synapse-associated protein expression in ASD animals. Collectively, these findings indicate that ENERGI, an orally administered AMPK activator, may offer a therapeutic advantage for ameliorating core ASD pathology with potentially fewer side effects compared to existing ASD medications, and highlight the potential of AMPK as a biomarker for ASD.

## Supplementary Information

Below is the link to the electronic supplementary material.

ESM 1 (PDF 8.67 MB)

## Data Availability

No datasets were generated or analysed during the current study.

## Abbreviations

aCSF: Artificial cerebrospinal fluid
AMPA: α-Amino-3-hydroxy-5-methyl-4-isoxazolepropionic acid
AMPK: AMP-activated protein kinase
ASD: Autism spectrum disorder
DCS: D-cycloserine
E: Embryonic day
fEPSP: Field excitatory postsynaptic potential
HFS: High-frequency stimulation
LFS: Low-frequency stimulation
LTD: Long-term depression
LTP: Long-term potentiation
mTOR: Mammalian target of rapamycin
NMDA: N-methyl-D-aspartate
PND: Postnatal day
PSD95: Postsynaptic density protein 95
VPA: Valproate
