# Supplementary material for: AMPK Activation by ENERGI Ameliorates Behavioral and Synaptic Deficits in a Mouse Model of Autism
Source: Mol Neurobiol. 2025 Nov 27;63(1):190. doi: 10.1007/s12035-025-05533-w (PMC12660476; doi:10.1007/s12035-025-05533-w)
Supplement: Supplementary file 1 — (PDF 8.67 MB) [file 12035_2025_5533_MOESM1_ESM.pdf]

Supplementary Information

Title: AMPK activation by ENERGI ameliorates behavioral and synaptic deficits in a mouse model of autism

Journal name: Molecular Neurobiology

Author names and affiliation:

Ming-Chia Chu[a], Chi-Chun Wu[a], Yueh-Jung Chung[a], Chieh-Yu Chang[a], Han-Fang Wu[b], Sze-Chi Tsai[a], Tzu-Ning Peng[a], Tzu-Jung Yang[a], Hui-Ching Lin[a,c,d,*]

[a]Department and Institute of Physiology, College of Medicine, National Yang Ming Chiao Tung University, Taipei, Taiwan;

[b]Department of Optometry, MacKay Medical College, New Taipei City, Taiwan;

[c]Ph.D. Program in Medical Neuroscience, College of Medical Science and Technology, Taipei Medical University, Taipei, Taiwan;

[d]Brain Research Center and Membrane Protein Structural Biology Research Center, National Yang Ming Chiao Tung University, Taipei, Taiwan.

Contact information of the corresponding author:

Hui-Ching Lin PhD, Department of Physiology, College of Medicine, National Yang Ming Chiao Tung University, Taipei 11221, Taiwan; Phone: +886-2-2826-7944; Fax: +886-2-2826-4049; E-mail: hclin7@nycu.edu.tw; huiching4372@gmail.com; ORCID: 0000-0002-1639-9293

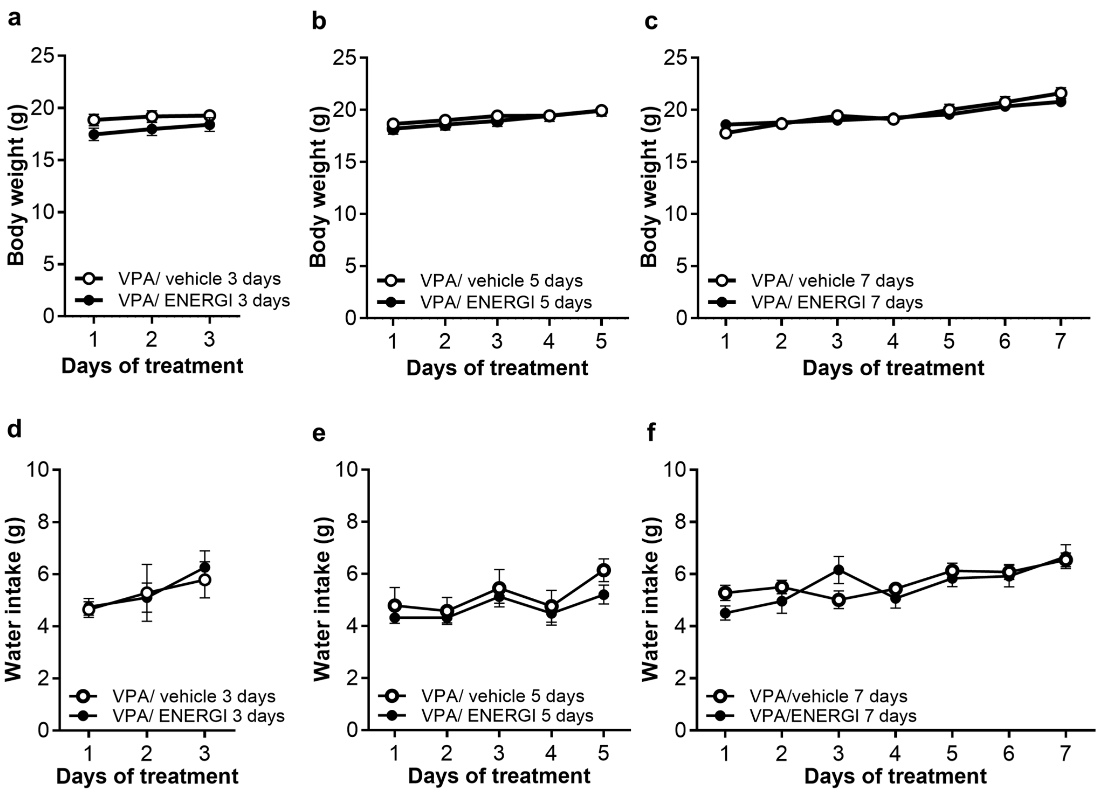

Fig. S1 Body weight and water intake of VPA-induced offspring during ENERGI treatment. (a−c) Daily body weight measured from VPA-induced offspring during vehicle or ENERGI treatment. (d−f) Daily water intake measured from VPA-induced offspring during vehicle or ENERGI treatment. Mice number: VPA/vehicle 3 days = 7, VPA/ENERGI 3 days = 14, VPA/vehicle 5 days = 7, VPA/ENERGI 5 days = 14, VPA/vehicle 7 days = 20, VPA/ENERGI 7 days = 17. Data shown as mean ± SEM. Two-way ANOVA followed by Bonferroni *post-hoc* tests

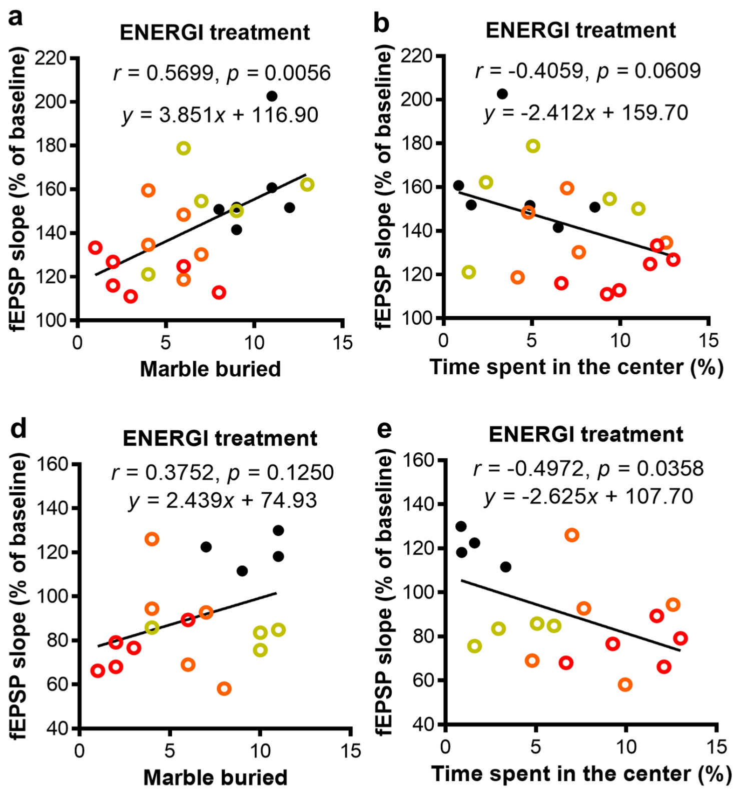

Fig. S2 Correlations between hippocampal synaptic plasticity and behavioral performance following ENERGI treatment in VPA-induced offspring. (a) Pearson correlation between fEPSP slope (50−60 min after HFS) and marble buried in the marble burying test. (b) Pearson correlation between fEPSP slope (50−60 min after HFS) and time spent in the center (%) in the open-field test. (c) Pearson correlation between fEPSP slope (50−60 min after HFS) and time in open arms (%) in the elevated plus maze test. (d) Pearson correlation between fEPSP slope (60−70 min after LFS) and marble buried in the marble burying test. (e) Pearson correlation between fEPSP slope (60−70 min after LFS) and time spent in the center (%) in the open-field test. (f) Pearson correlation between fEPSP slope (60−70 min after LFS) and time in open arms (%) in the elevated plus maze test. Data presented as scatter plots with linear regression lines using Pearson correlation and linear regression analysis

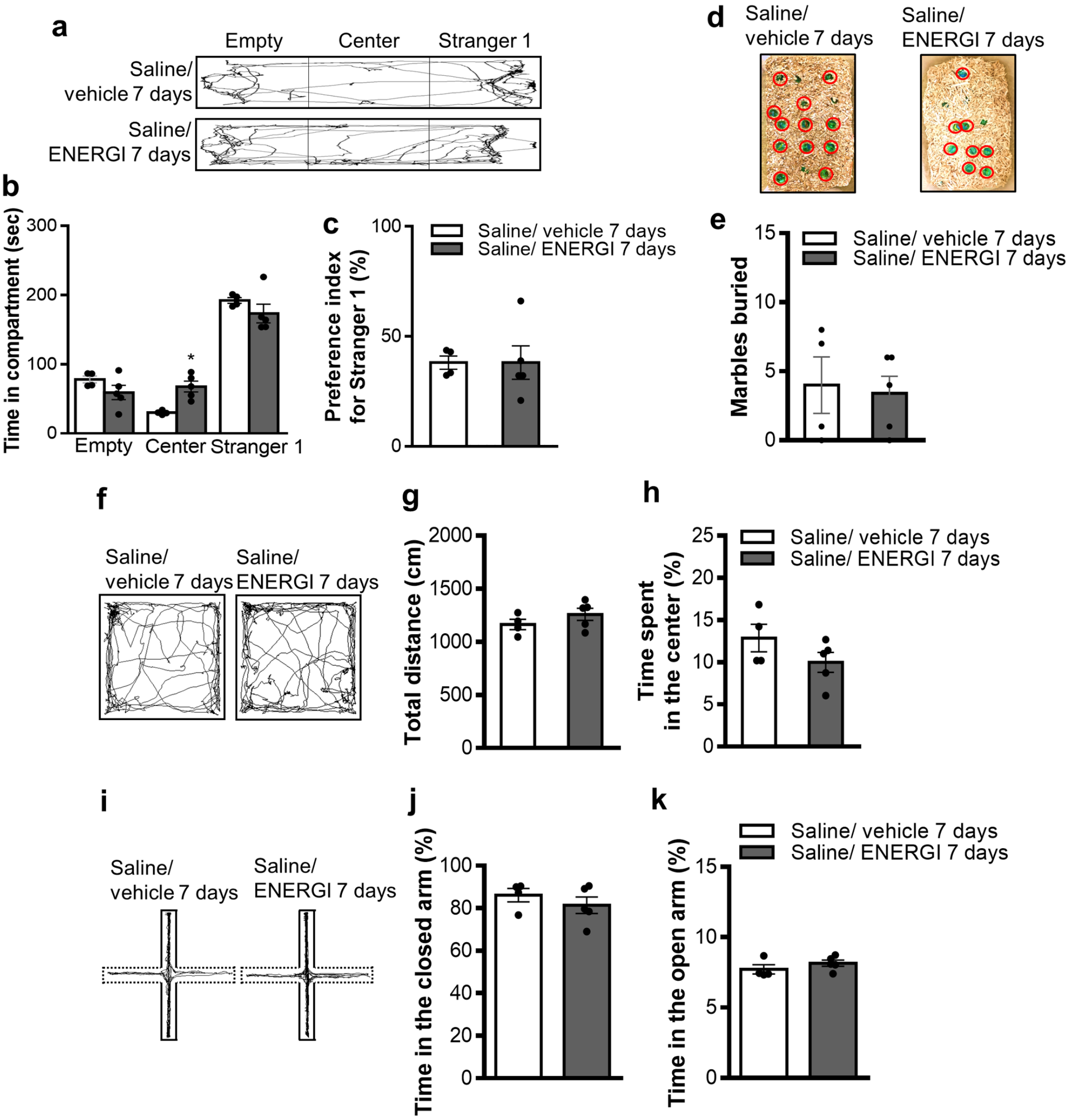

Fig. S3 Effects of 7-day ENERGI treatments on ASD-related behaviors in control offspring. (a–b) Representative traces (a) and time exploring three compartments (b) during the three-chamber sociability test after ENERGI, or vehicle treatment in control offspring. (c) Preference index calculated based on chamber duration in the three-chamber sociability test after ENERGI, or vehicle treatment in control offspring. (d–e) Representative image at the end of the experiment (d) and the number of marbles buried (e) during the marble burying test after ENERGI, or vehicle treatment in control offspring. (f–h) Representative traces (f), total distance (g), and time spent in the center of the open field (h) during the open field test after ENERGI, or vehicle treatment in control offspring. (i–k) Representative traces (i), and time spent in the closed arm (j) and open arm (k) during the elevated plus maze test after ENERGI, or vehicle treatment in control offspring. Mice number: Saline/ vehicle 7 days = 4, Saline/ ENERGI 7 days = 5. Data shown as mean ± SEM. *p <0.05 vs Saline/vehicle using Student's t-test or two-way ANOVA followed by Bonferroni *post-hoc* tests

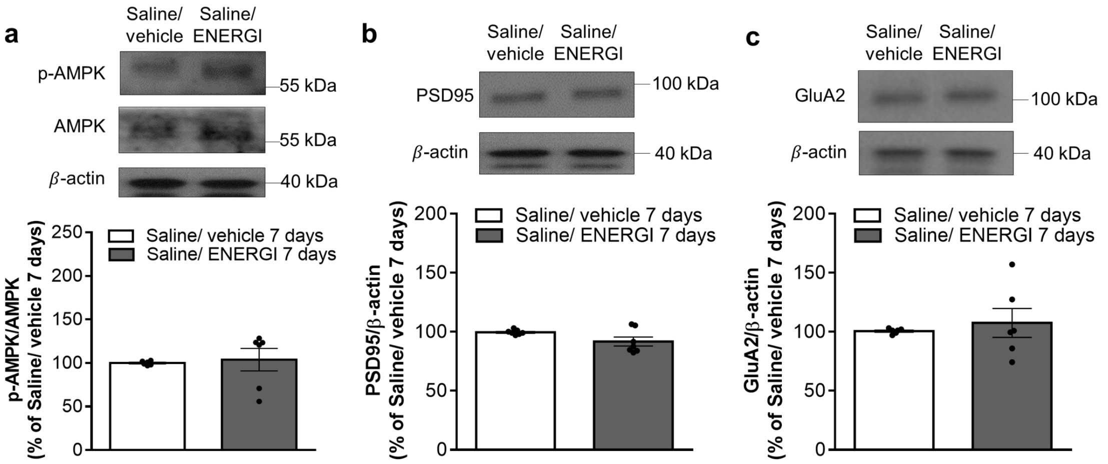

Fig. S4 Effects of 7-day ENERGI treatments on AMPK and synapse-associated proteins in control offspring. (a–c) Representative immunoblot and summary bar graph showing the phosphorylation levels of AMPK (a), levels of PSD95 (b), and synaptic levels of GluA2 (c) in the hippocampus after 7-day ENERGI, or vehicle treatment in control offspring. For p-AMPK, mice number = 6 in each group. For PSD95, mice number = 7 in each group. For GluA2, mice number = 6 in each group. Data shown as mean ± SEM. Student's *t*-test

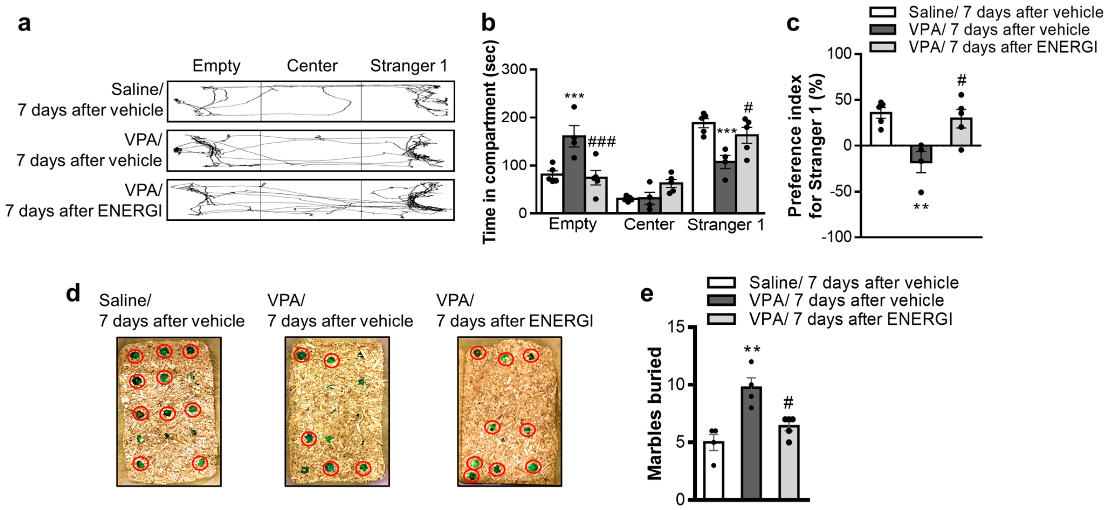

Fig. S5 Effects of ENERGI on ASD-related behaviors 7 days after treatments in VPA-induced offspring. (a–b) Representative traces (a) and time exploring three compartments (b) during the three-chamber sociability test 7 days after ENERGI, or vehicle treatment in control and VPA-induced offspring. (c) Preference index calculated based on chamber duration in the three-chamber sociability test 7 days after ENERGI, or vehicle treatment in control and VPA-induced offspring. (d–e) Representative image at the end of the experiment (d) and the number of marbles buried (e) during the marble burying test 7 days after ENERGI, or vehicle treatment in control and VPA-induced offspring. Saline/7 days after vehicle: n=4–5. VPA/7 days after vehicle: n=4. VPA/7 days after ENERGI: n=5. Data shown as mean ± SEM. $^{**}p < 0.01$, $^{***}p < 0.001$ vs Saline/7 days after vehicle; $^{\#}p < 0.05$ vs. VPA/7 days after vehicle using one-way ANOVA or two-way ANOVA followed by Bonferroni *post-hoc* tests

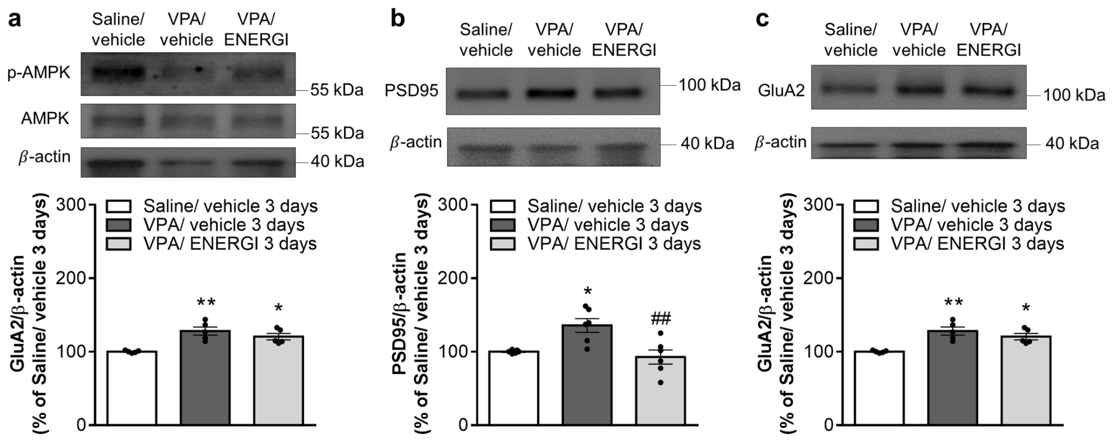

Fig. S6 Effects of 3-day ENERGI treatments on AMPK and synapse-associated proteins in VPA-induced offspring. (a–c) Representative immunoblot and summary bar graph showing the phosphorylation levels of AMPK (A), levels of PSD95 (b), and synaptic levels of GluA2 (c) in the hippocampus after 3-day ENERGI, or vehicle treatment in control and VPA-induced offspring. For p-AMPK, mice number = 4 in each group. For PSD95, mice number = 6 in each group. For GluA2, mice number = 5 in each group. Data shown as mean ± SEM. *p <0.05, **p <0.01, ***p <0.001 vs Saline/vehicle; $^{\#}p$ <0.05, $^{\#\#}p$ <0.01 vs. VPA/vehicle using one-way ANOVA followed by Bonferroni *post-hoc* tests

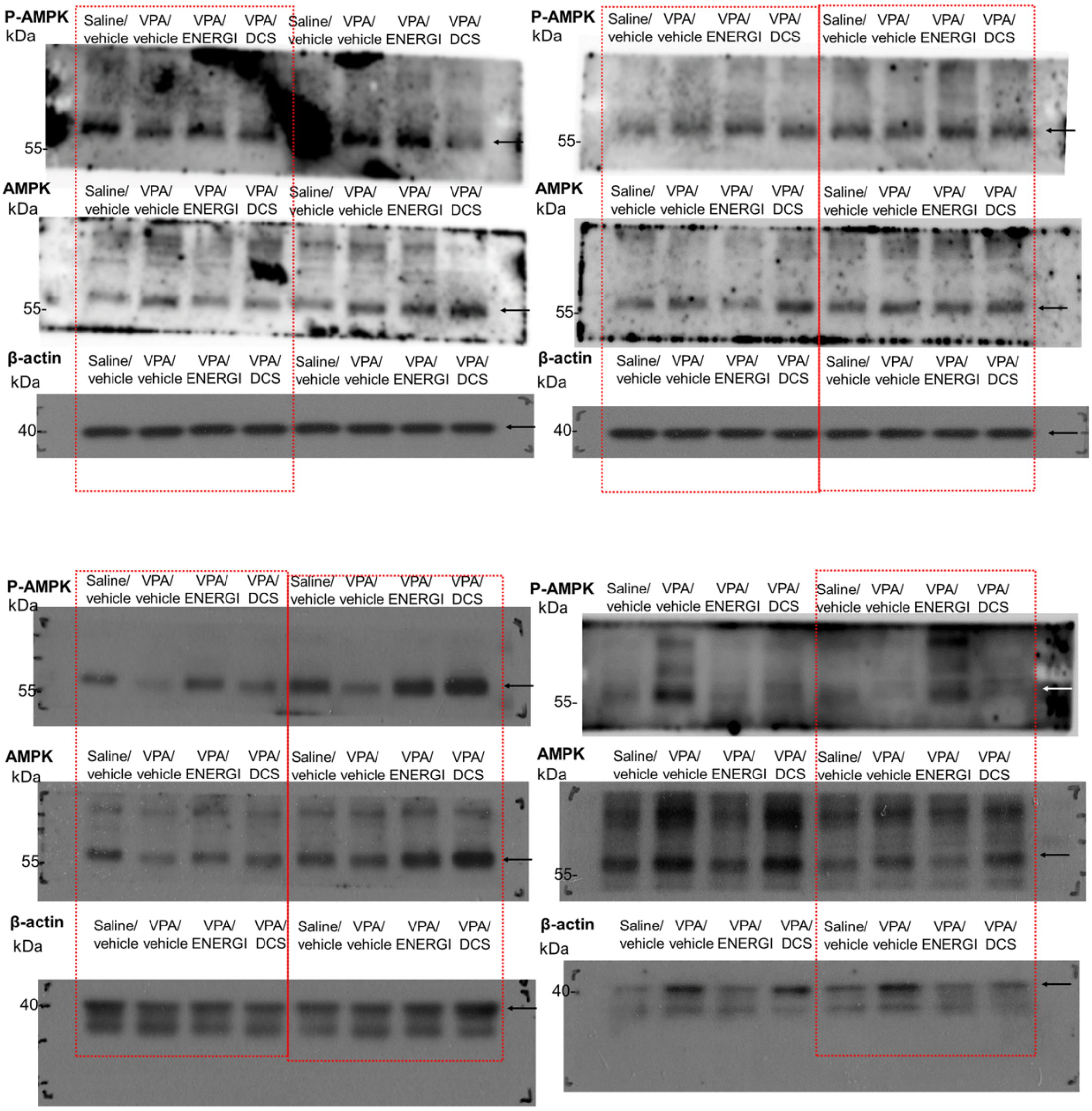

Fig. S7 Full blot images for Figure 9A

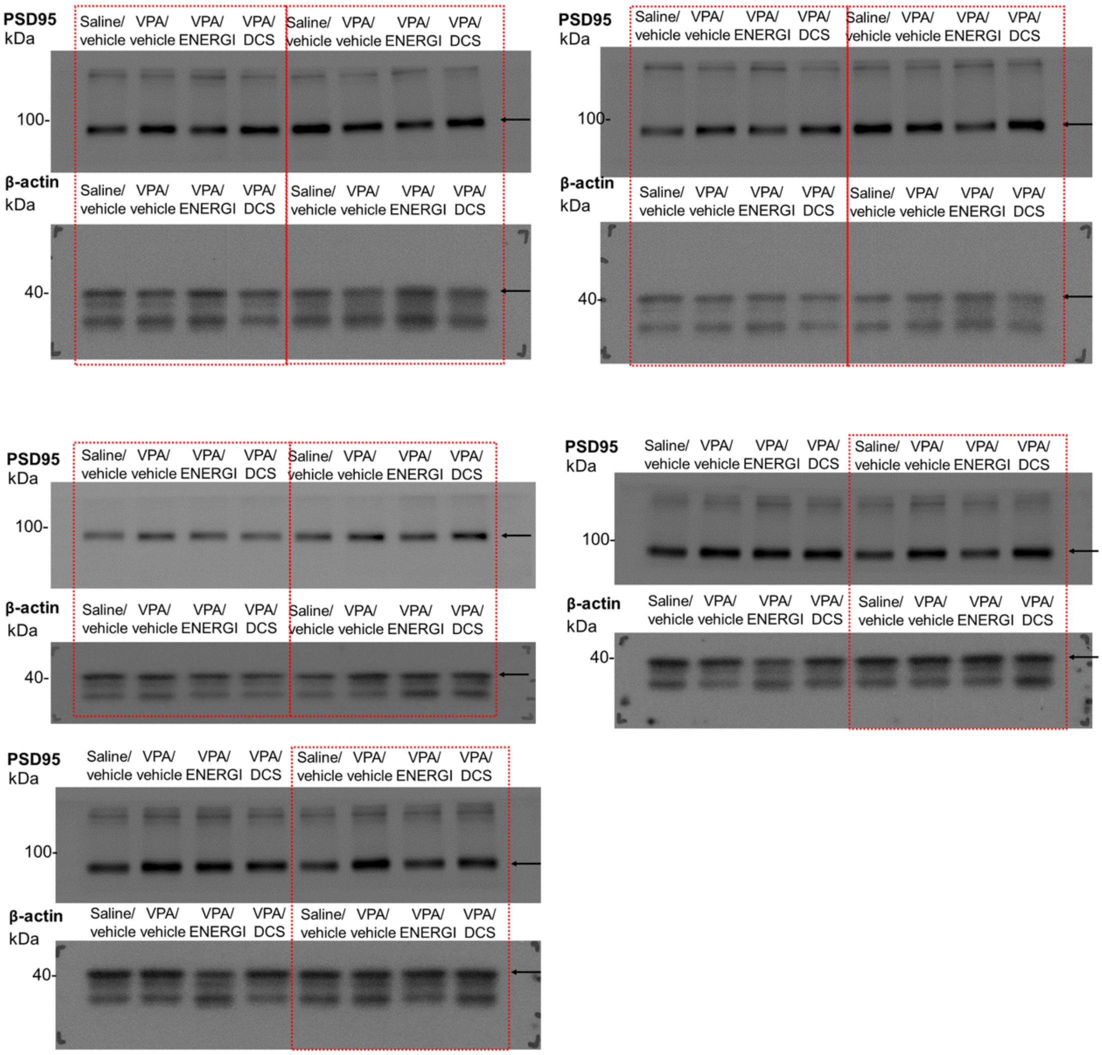

Fig. S8 Full blot images for Figure 9B

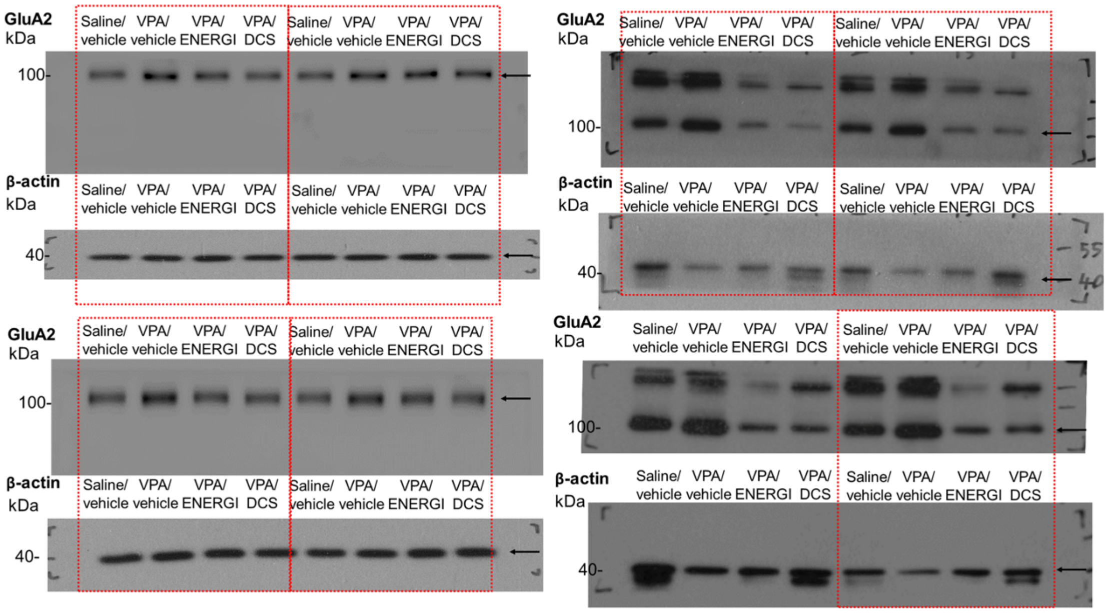

Fig. S9 Full blot images for Figure 9C

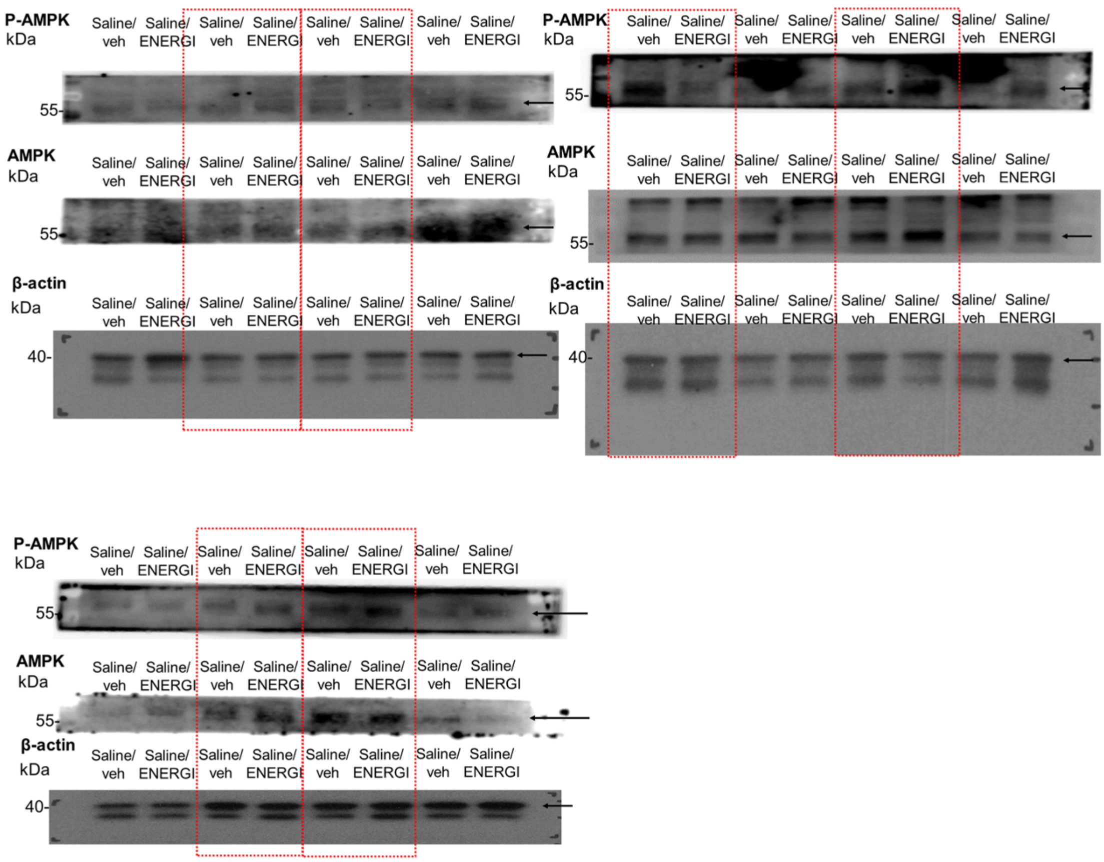

Fig. S10 Full blot images for Supplementary Figure 4A

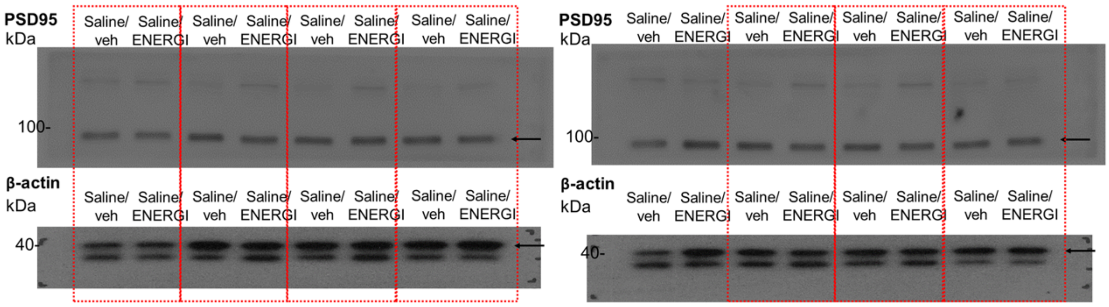

Fig. S11 Full blot images for Supplementary Figure 4B

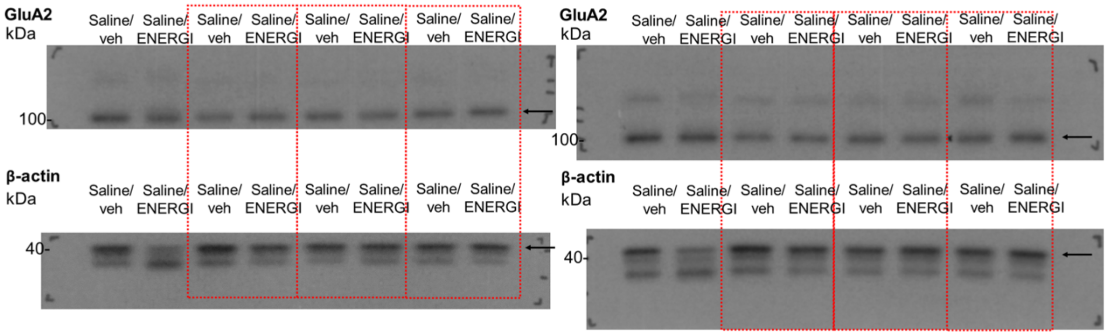

Fig. S12 Full blot images for Supplementary Figure 4C

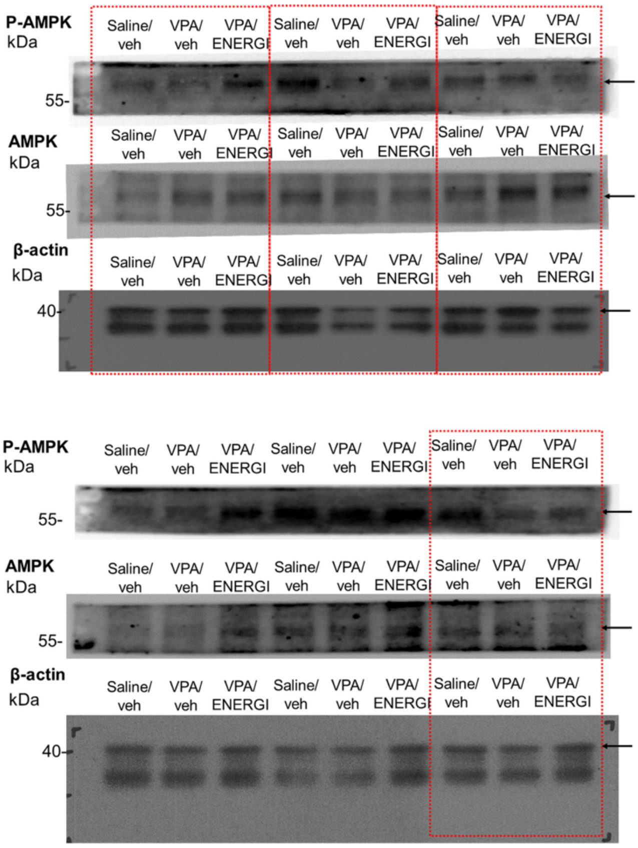

Fig. S13 Full blot images for Supplementary Figure 6A

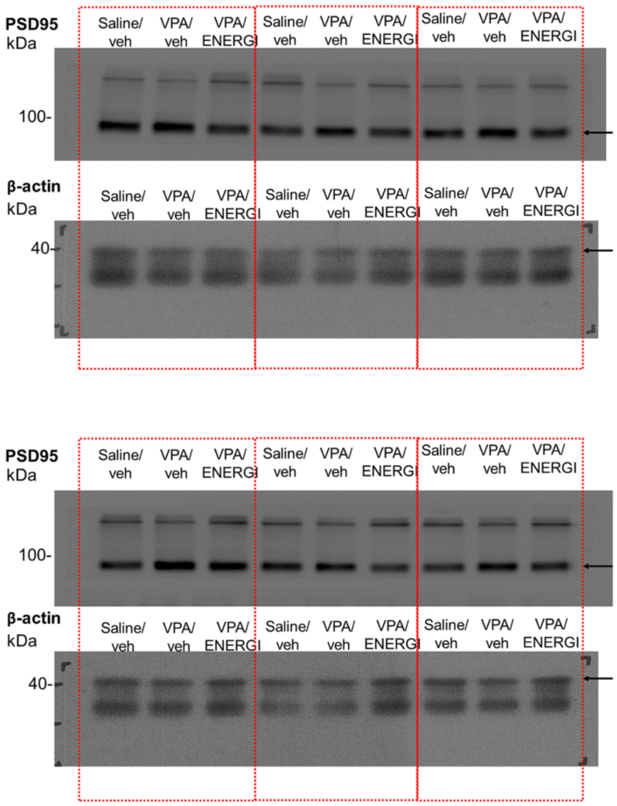

Fig. S14 Full blot images for Supplementary Figure 6B

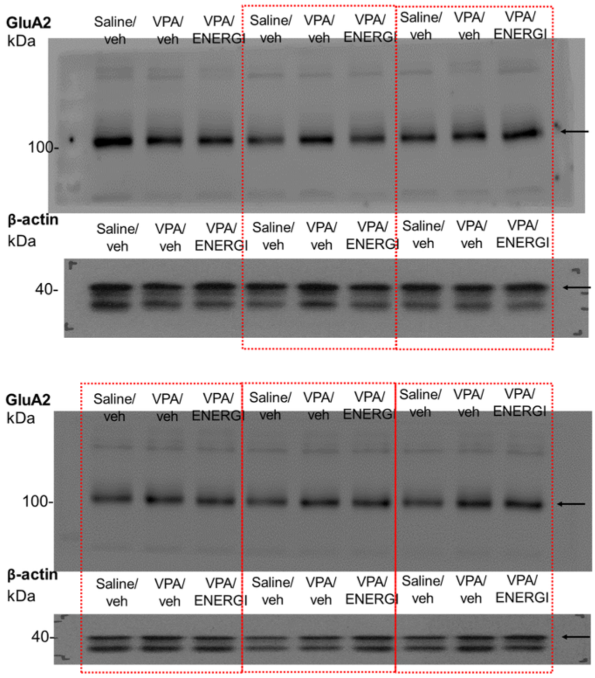

Fig. S15 Full blot images for Supplementary Figure 6C
